# From the motor cortex to the movement and back again

**DOI:** 10.1371/journal.pone.0179288

**Published:** 2017-06-20

**Authors:** Wondimu W. Teka, Khaldoun C. Hamade, William H. Barnett, Taegyo Kim, Sergey N. Markin, Ilya A. Rybak, Yaroslav I. Molkov

**Affiliations:** 1 Indiana University–Purdue University at Indianapolis, Indianapolis, Indiana, United States of America; 2 Drexel University College of Medicine, Philadelphia, Pennsylvania, United States of America; 3 Georgia State University, Atlanta, Georgia, United States of America; Duke University, UNITED STATES

## Abstract

The motor cortex controls motor behaviors by generating movement-specific signals and transmitting them through spinal cord circuits and motoneurons to the muscles. Precise and well-coordinated muscle activation patterns are necessary for accurate movement execution. Therefore, the activity of cortical neurons should correlate with movement parameters. To investigate the specifics of such correlations among activities of the motor cortex, spinal cord network and muscles, we developed a model for neural control of goal-directed reaching movements that simulates the entire pathway from the motor cortex through spinal cord circuits to the muscles controlling arm movements. In this model, the arm consists of two joints (shoulder and elbow), whose movements are actuated by six muscles (4 single-joint and 2 double-joint flexors and extensors). The muscles provide afferent feedback to the spinal cord circuits. Cortical neurons are defined as cortical "controllers" that solve an inverse problem based on a proposed straight-line trajectory to a target position and a predefined bell-shaped velocity profile. Thus, the controller generates a motor program that produces a task-specific activation of low-level spinal circuits that in turn induce the muscle activation realizing the intended reaching movement. Using the model, we describe the mechanisms of correlation between cortical and motoneuronal activities and movement direction and other movement parameters. We show that the directional modulation of neuronal activity in the motor cortex and the spinal cord may result from direction-specific dynamics of muscle lengths. Our model suggests that directional modulation first emerges at the level of muscle forces, augments at the motoneuron level, and further increases at the level of the motor cortex due to the dependence of frictional forces in the joints, contractility of the muscles and afferent feedback on muscle lengths and/or velocities.

## Introduction

Even simple arm movements such as reaching require complex interactions among the central and peripheral nervous systems, and skeletal muscles to generate the intended arm movements. Over three decades, a lot of effort has been put into understanding neural mechanisms controlling reaching movements [1–4]. Reaching is broadly defined as the arm’s movement starting at some initial position in space and ending at a target position. In experiments, unperturbed reaching movement usually occurs along a straight-line trajectory with a bell-shaped velocity profile [5]. Dynamically, reaching movements result from complex concurrent or sequential activation patterns of multiple muscles used to accelerate and then, slow down and stop the arm along the intended trajectory. To generate the required muscle activation patterns, the motor cortex needs to solve a corresponding “inverse problem” and, based on this solution, provide the appropriate dynamical inputs to the spinal circuits [6–8].

In reaching tasks, the relationships between neuronal activity in the motor cortex and movement parameters have been widely debated, and remain controversial [9–12]. It has been suggested that the neuronal activity in the primary motor cortex (M1) encodes such movement parameters as direction [2, 12–14], hand position [15–18], velocity [19], acceleration [20], and reaching distance [13, 18]. However, other studies have argued that neural activity in the motor cortex correlates with kinetic variables, such as forces and torques [21, 22]. In 1982, Georgopoulos et al. demonstrated for the first time a correlation between neuronal activity in the motor cortex and the direction of reaching movement [2]. They showed that the average firing rate of M1 neurons during reaching movements varied with the direction of movement, and that each M1 neuron had a preferred direction (PD) for which its average firing rate was maximal. Since then, directional tuning has been ubiquitously considered as a key property of neural activity in the motor cortex. However, it remains controversial whether directional preference is the fundamental property of cortical neurons or a side effect of muscle activity or other movement features [11, 23–27].

Although many movement parameters correlate with cortical activity, the cause of these correlations is still not clear. For example, it has been suggested that the directional sensitivity of cortical neurons is the result of a specific organization of inhibitory interactions within and between neuronal columns in the motor cortex [28, 29]. A competing viewpoint is that cortical activity is related to the activity of corresponding muscles that have anisotropic properties and thus, form cortical directional tuning [27, 30–32]. Moreover, the contribution of the spinal cord circuitry to directional modulation is not well understood.

Mathematical models have been used to better understand the relationship between the activity of neurons in the motor cortex and movement parameters during reaching [32–34]. However, previous models did not consider the spinal cord network and/or length/velocity-dependence of contractility of the muscle controlling the movement. In the present study, we have developed an integrative mathematical model of a motor control system that incorporates cortical neuronal populations, complex spinal neural circuits controlling arm muscles and receiving afferent feedback from them, and a two-joint arm actuated by these muscles that performs reaching movements in a 2D space. The arm model includes the shoulder and elbow joints whose movements are generated by pairs of flexor and extensor muscles controlling a single joint and two bi-articular flexor and extensor muscles controlling both joints at the same time. The arm model is very similar to the model developed by Lillicrap and Scott [32]. Unlike Lillicrap and Scott who used a complex cortical neuronal network including a learning system to control the arm, we focused on a spinal cord network which receives aggregate signals from six supraspinal neuronal pools. The cortical activity (hereinafter referred to as a motor program) was calculated by solving an inverse problem based on a straight-line trajectory to a target position and a predefined bell-shaped velocity profile. This approach allowed us to generate and analyze a variety of activity profiles of cortical neurons that may be used to perform movements to the same or different target positions. We evaluated our findings in the context of different theoretical hypotheses concerned with the neural control for reaching [30–32]. Particularly, Lillicrap and Scott [32] showed that limb geometry, intersegmental dynamics, and the force length and force velocity properties of muscle are the main causes of directional preferences of cortical neurons during reaching movements. In agreement with Lillicrap and Scott, our results show that the muscle length and velocity dependent contractile components of the muscle forces are the root causes of the directional preference of spinal motoneurons. Furthermore, we showed that afferent feedback has significant impact on the directional behavior of cortical neurons and proposed that directional dependence of the mean firing rates of M1 neurons primarily results from afferent feedback signals carrying information muscle lengths and velocities. Moreover, our results show that the spinal cord circuit may play a comparable or even more significant role in directional tuning of M1 neurons than the contractile components of the muscle forces. The model reveals the mechanisms by which directionally indifferent torques in the arm joints imply directionally tuned cortical activity.

## Results

**Fig 1:**
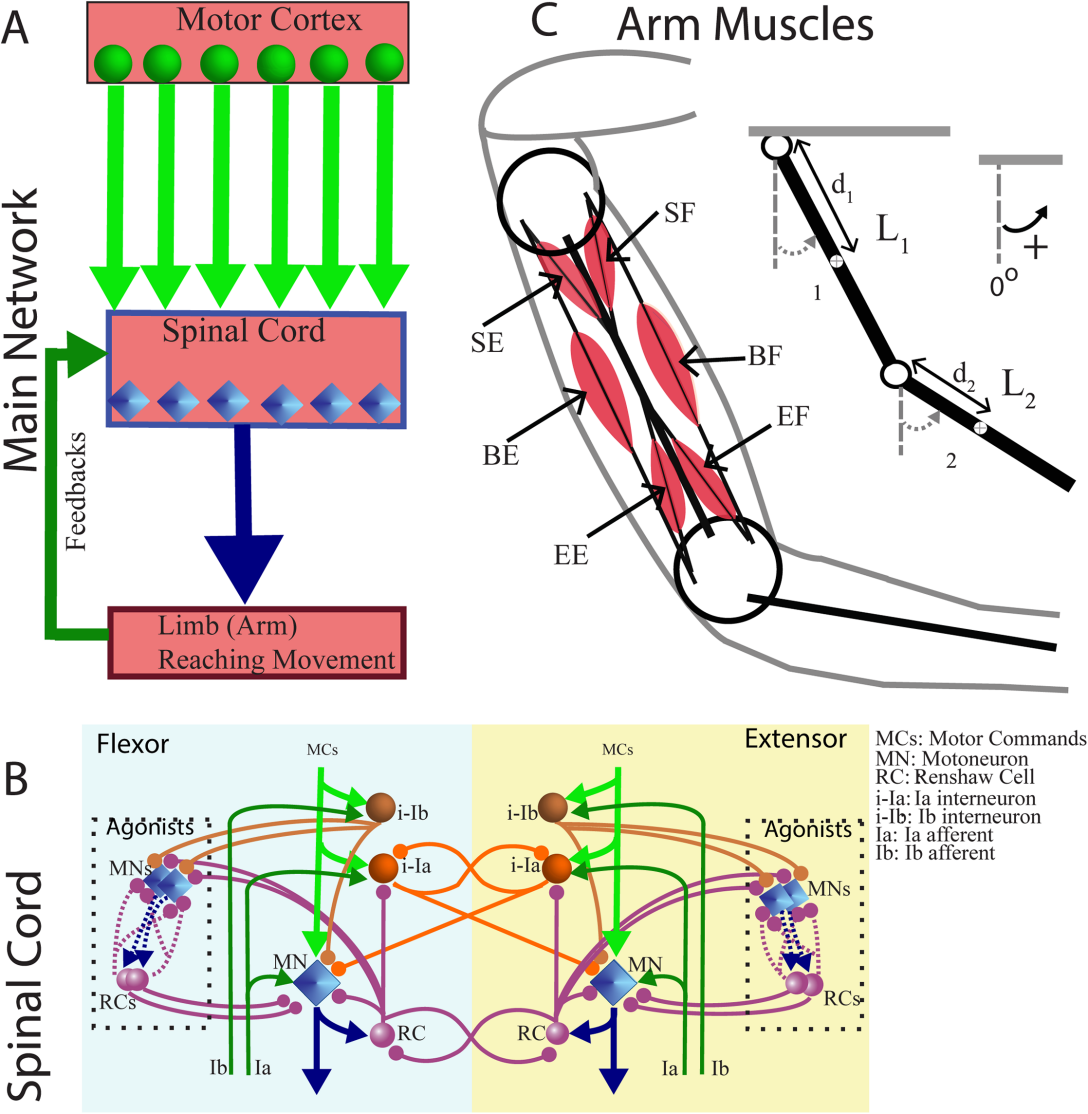
Model structure. **(A)** The motor cortex sends motor signals to the spinal cord neuronal network which sends its outputs to the muscles. The spinal cord combines motor signals with afferent feedback to generate the motorneuron outputs. **(B)** Organization of interconnections between Renshaw cells (RC), motoneurons (MN) and other interneurons in the spinal cord network. Motoneurons send their outputs to their corresponding arm muscles. Ia and Ib inputs are the feedback signals from the muscles. **(C)** A 2-joint arm with six muscles: four major flexor and extensor muscles about the shoulder and elbow joints, and two biarticular muscles controlling both shoulder and elbow joints. SF, EF and BF represent shoulder, elbow and biarticular flexors, and SE, EE and BE represent shoulder, elbow and biarticular extensors, respectively.

The model presented here comprises three main modules: Arm, Spinal Cord, and Motor Cortex (Fig 1A). The Arm module is modeled as a mechanical system of two rigid segments and two joints (shoulder and elbow) controlled by six Hill-type muscles, namely: the shoulder flexor (SF) and extensor (SE), the elbow flexor (EF) and extensor (EE), and the two-joint extensor (BE) and flexor (BF) (see Fig 1C). Arm movements are restricted to a horizontal plane and are produced by coordinated activation of the muscles. The Spinal Cord module has neural circuits that receive descending signals from the Motor Cortex, and relay them through spinal motoneurons to the corresponding muscles. The Spinal Cord neurons receive afferent feedback from the muscles and form local reflex circuits. These include (1) monosynaptic excitation of homonymous motoneurons by Ia muscle afferents; (2) reciprocal inhibition between the antagonistic flexor and extensor motoneurons via Ia interneurons receiving Ia afferents; (3) non-reciprocal inhibition of homonymous and synergistic motoneurons by Ib muscle afferents via the Ib interneurons; and (4) recurrent inhibition of motoneurons via corresponding Renshaw cells (see Fig 1B). The Motor Cortex module projects the activity of six cortical neuronal populations (M1-N) down to six motoneuron pools in the Spinal Cord to control movements of the arm (see Fig 1A and 1B). This descending command quantitatively represents the aggregate cortical input rather than specific mono- or poly-synaptic projections. A detailed description of the model including mathematical formulations is provided in the Methods section.

### Directional modulation of cortical neurons

**Fig 2:**
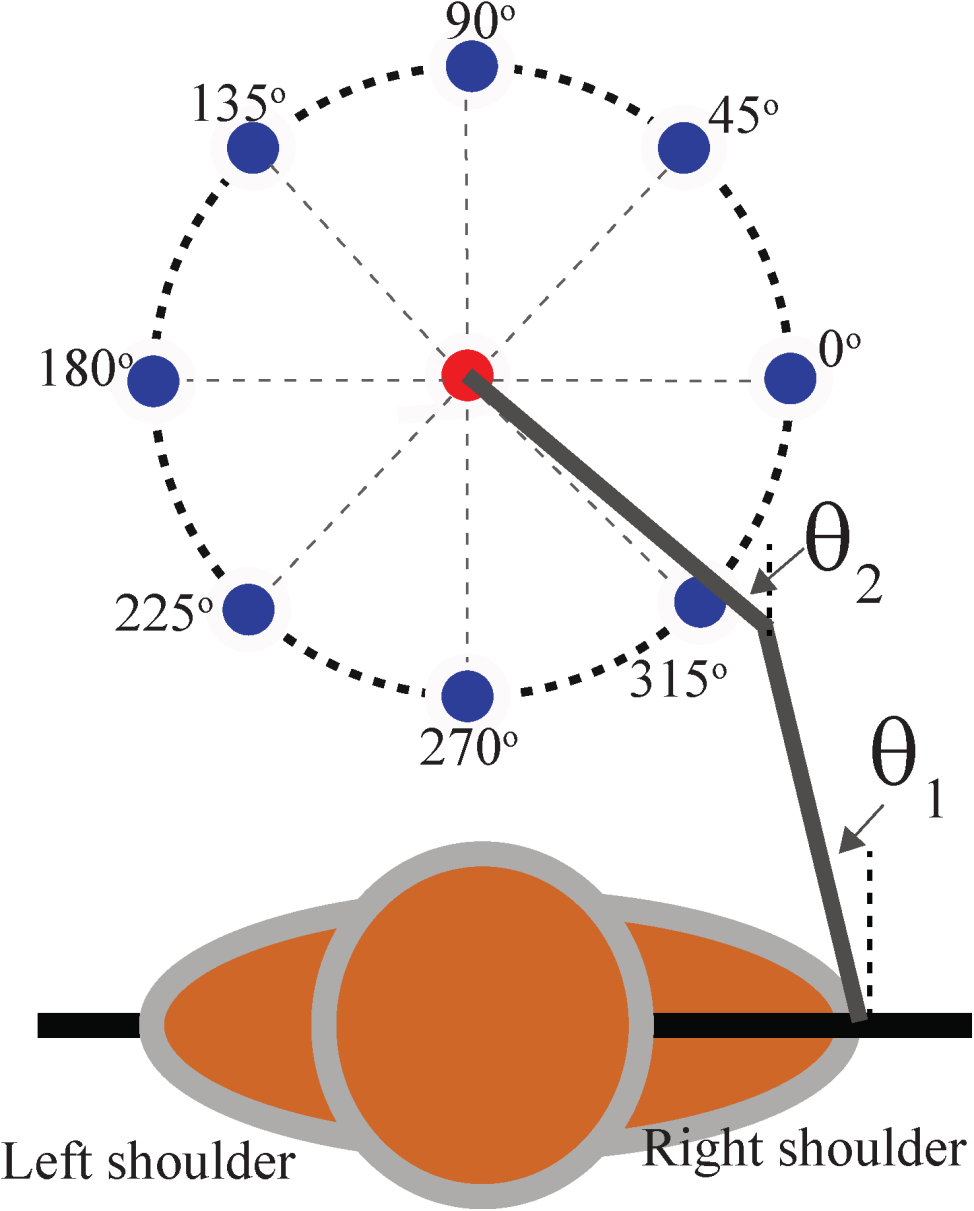
Setup of the center-out reaching task in 8 directions with a simulated arm model. The arm model performs movements from the initial position (center, red point) to one of the 8 peripheral target positions (outer, dark blue points). For all simulations, except where noted, the reaching distance, equal to the radius of the circle, was fixed to 0.2 meters, and the reaching time was fixed to 1 second. Angles θ_**1**_ and θ_**2**_ represent the shoulder and elbow joint angles, respectively, with respect to the vertical axis, similar to Fig 1C.

**Fig 3:**
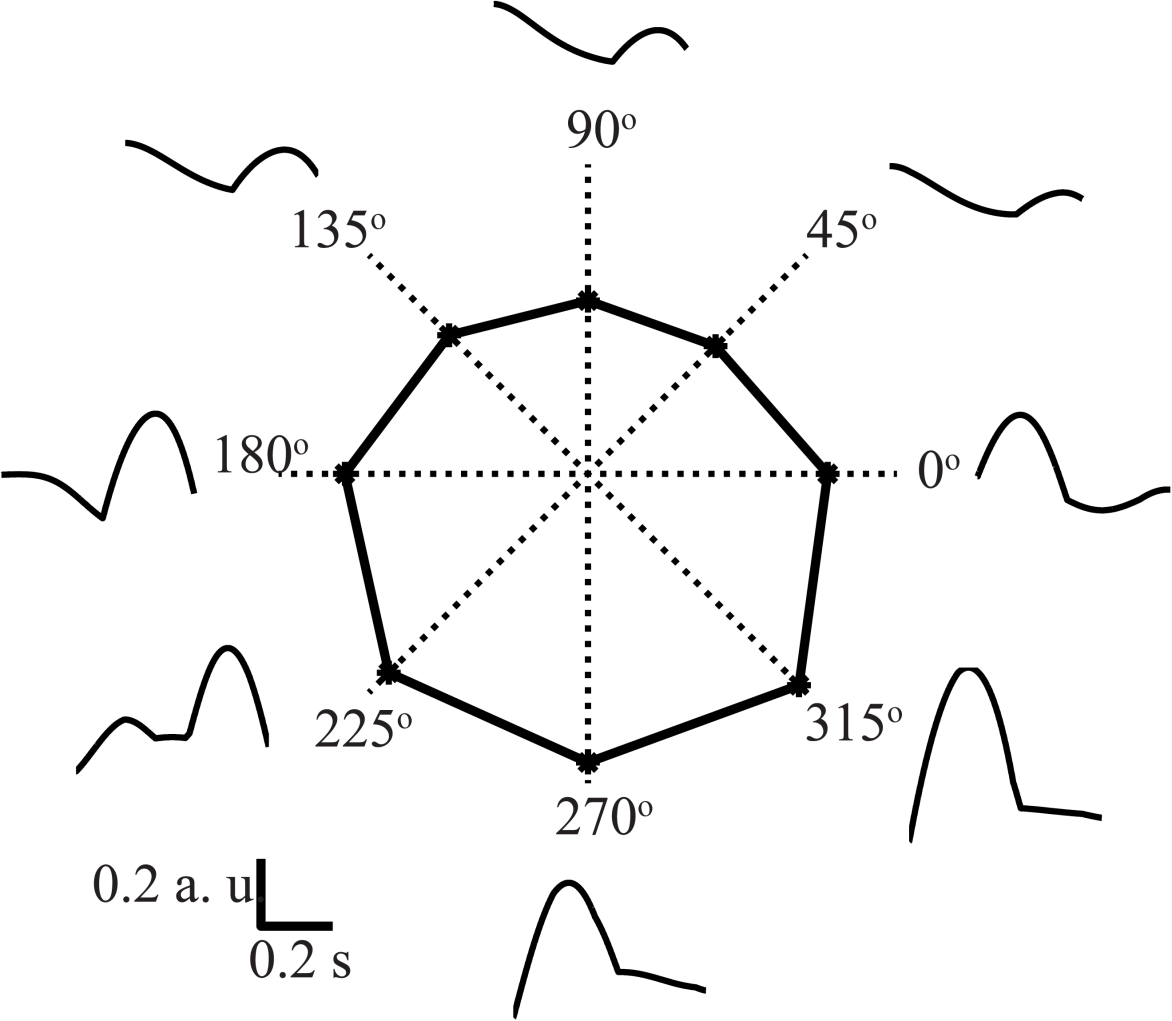
Directionally modulated cortical activity. Model performance: activity of a population of cortical neurons (a single simulation computed based on Eq 16) that controls the 1-joint elbow flexor. For the center-out reaching movement in 8 directions (45º intervals), the activity patterns of this neuronal pool are shown by black solid curves, demonstrating the highest and lowest mean firing rates in opposite directions (180º apart). The average firing rate (solid polygon) is highest for the 270º direction.

Using our mathematical model, we simulated center-out reaching tasks in 8 different directions and analyzed the activity of each M1 neuron (Fig 2). We found that the firing rate of these neurons depended on the movement direction, such that it was maximal for one direction (preferred direction, PD, denoted by *Θ*_*PD*_), and minimal for the opposite direction (anti-PD, 180^o^ apart from *Θ*_*PD*_). Fig 3 shows an example of the neural activity of a population of M1 cortical neurons that projects to the elbow flexor motoneuron and is therefore responsible for the contraction of the elbow flexor muscle during reaching movements in all 8 directions. The activity profiles (outer traces in Fig 3) show dynamical and quantitative changes when the movement direction changes. This particular population of M1 neurons showed the highest amplitude of activity when reaching movements were made in the 270^o^-315^o^ direction, and the lowest amplitude of activity when reaching movements were made in the 90^o^-135^o^ direction. The firing rate of this particular neuron averaged over the entire reaching movement exhibited the highest value for the 270^o^ direction and the lowest value for the 90^o^ direction (polar curve in the center of Fig 3). This type of directionality has been previously observed in the experimental studies; see an example of the spiking activity of a cortical neuron recorded during a center-out reaching task performed by a primate in 8 different directions by Moran and Schwartz (Fig 6 in [19]). This example is qualitatively similar to our simulation results in terms of both average firing rate and changes in pattern.

**Fig 4:**
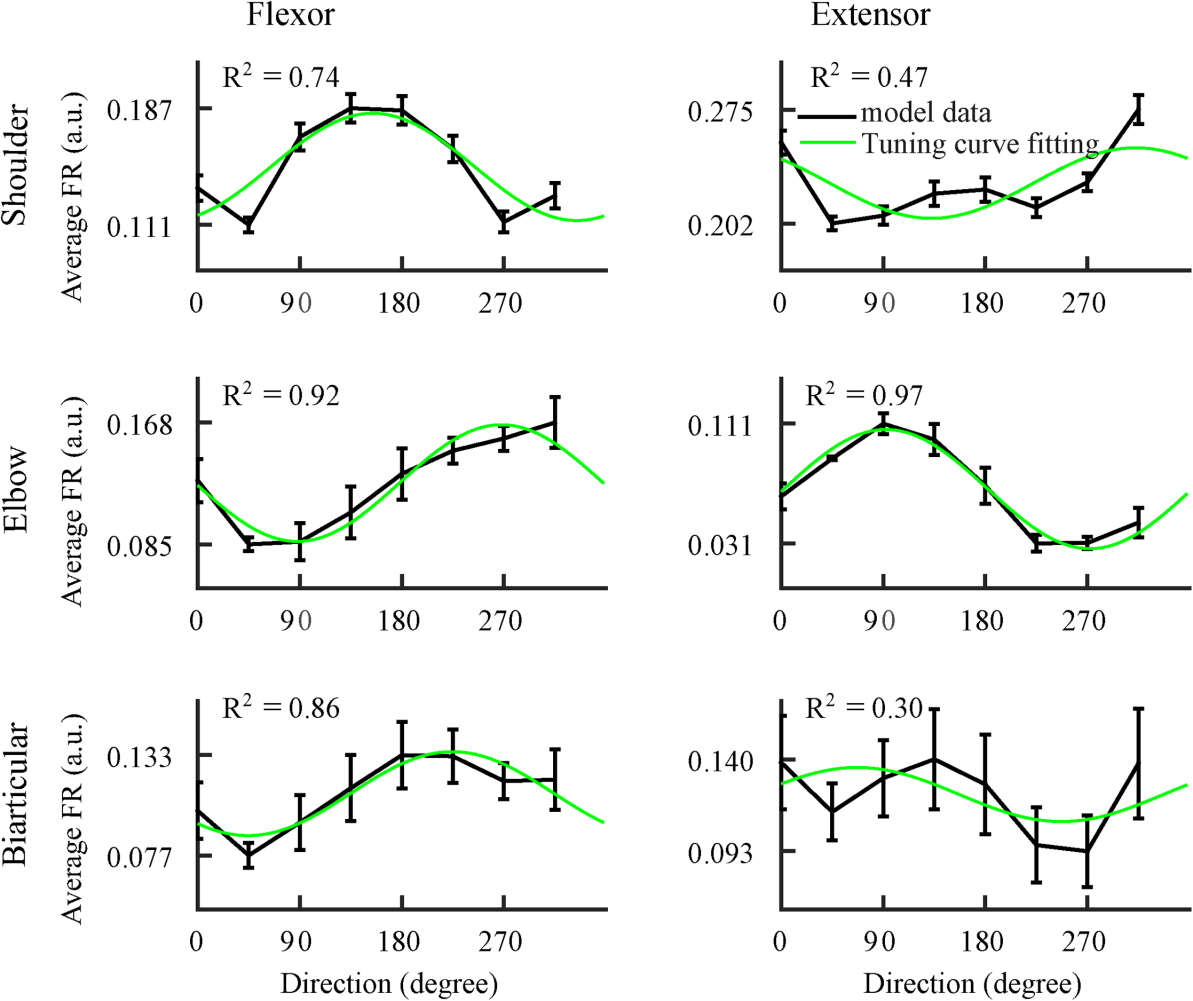
Cortical activity fits well to the cosine tuning curve. Average cortical activity (black, ± SD) of neurons controlling each of the 6 arm muscles fit to a cosinusoidal tuning curve (green curve). Four of the six cortical activities are strongly directionally tuned (coefficient of determination R^2^ > 0.7). Error bars show standard errors across trials with randomly chosen motor strategy (see text for more details). Simulation results are in agreement with experimental studies (Georgopoulos, Kalaska et al. [2], Fu, Suarez et al. [13], Moran and Schwartz [19]).

Due to the redundancy of arm biomechanics, a continuum of different muscle activation patterns may result in the exactly same movement (see Methods). To examine similarity of directional properties among different possible motor programs, we performed 50 center-out reaching trials while randomly selecting the torque distribution parameter (*d*) between mono- and bi-articular muscles (see Methods) in the range (0.5 ~ 1) using a uniform probability distribution for the 8 movement directions. All six M1 neurons–corresponding to SF, SE, EF, EE, BF, and BE–showed similar changes in their activity profiles and averages in conjunction with the change in movement direction, regardless of the torque distribution. We computed single-trial averages of the M1 neuron's firing rate over the duration of each movement. For each reaching direction, we computed the mean of the single-trial averages to produce a multi-trial average. We found that multi-trial averages varied in an orderly fashion with movement direction (Fig 4, black curves). Multi-trial averages were maximal for one unique movement direction, which was the PD for that particular M1 neuron. Each M1 neuron had a PD distinct from the other five neurons. The multi-trial activity of four M1 neurons (corresponding to SF, EF, EE, and BF) demonstrated coefficients of determination (*R*^*2*^, a measure of the precision of the regression fit) greater than 0.7 when fitted to cosine tuning curves (Fig 4, green curves), implying that the activity of these four M1 neurons was strongly correlated with movement direction. However, the two M1 neurons corresponding to SE and BE did not demonstrate as good a fit with the cosine-shaped tuning curve (*R*^*2*^ = 0.47 and 0.30, respectively), suggesting weaker directional modulation. The M1 neurons’ PDs, as determined by their cosine tuning curves, were 154^o^, 312^o^, 268^o^, 92^o^, 225^o^ and 67^o^, corresponding to SF, SE, EF, EE, BF and BE, respectively. Activity of M1 neurons, corresponding to antagonist flexors and extensors, exhibited opposite PDs separated by approximately 180^o^ (Fig 4). We have found that there is no change in preferred directions when the model uses only the shoulder and elbow muscles (by removing the biarticular muscle), which is in an agreement with Lillicrap and Scott [32]. However, the simulations show that biarticular muscles have significant impact on the coefficients of determination of the tuning curves.

### Directional modulation of spinal motoneurons and afferent feedback

**Fig 5:**
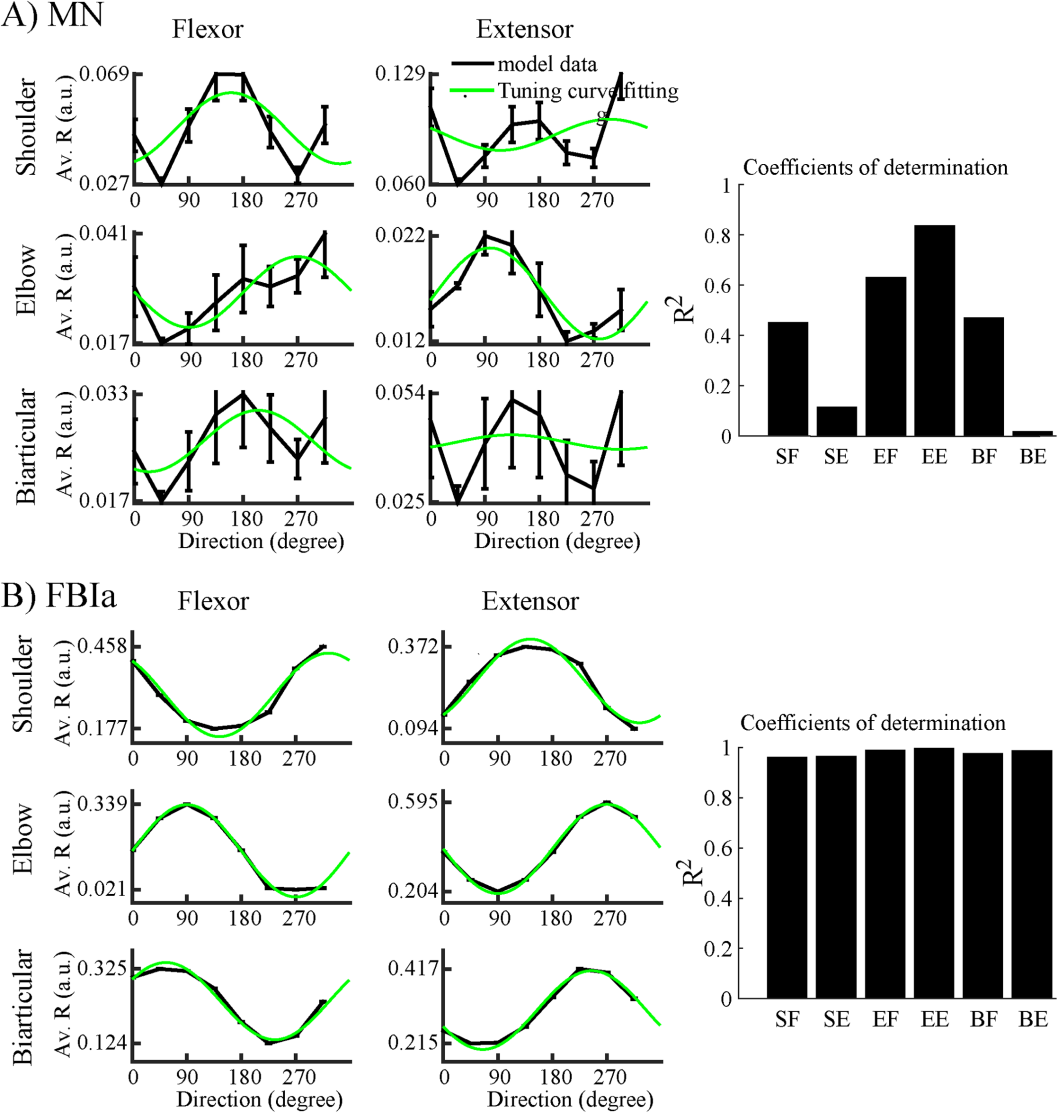
Directional modulation of spinal motoneurons and Ia afferents. **(A)** The pools of spinal motoneurons are sensitive to movement directions. The average responses (Av. R) of populations of these motoneurons (black, ± SD) are fitted with cosine tuning curves (green curve) and less directionally tuned than primary cortical neurons. **(B)** Activity of Ia afferent (FBIa) is strongly directionally turned (coefficient of determination R^2^ > 0.9) for all six muscles.

**Fig 6:**
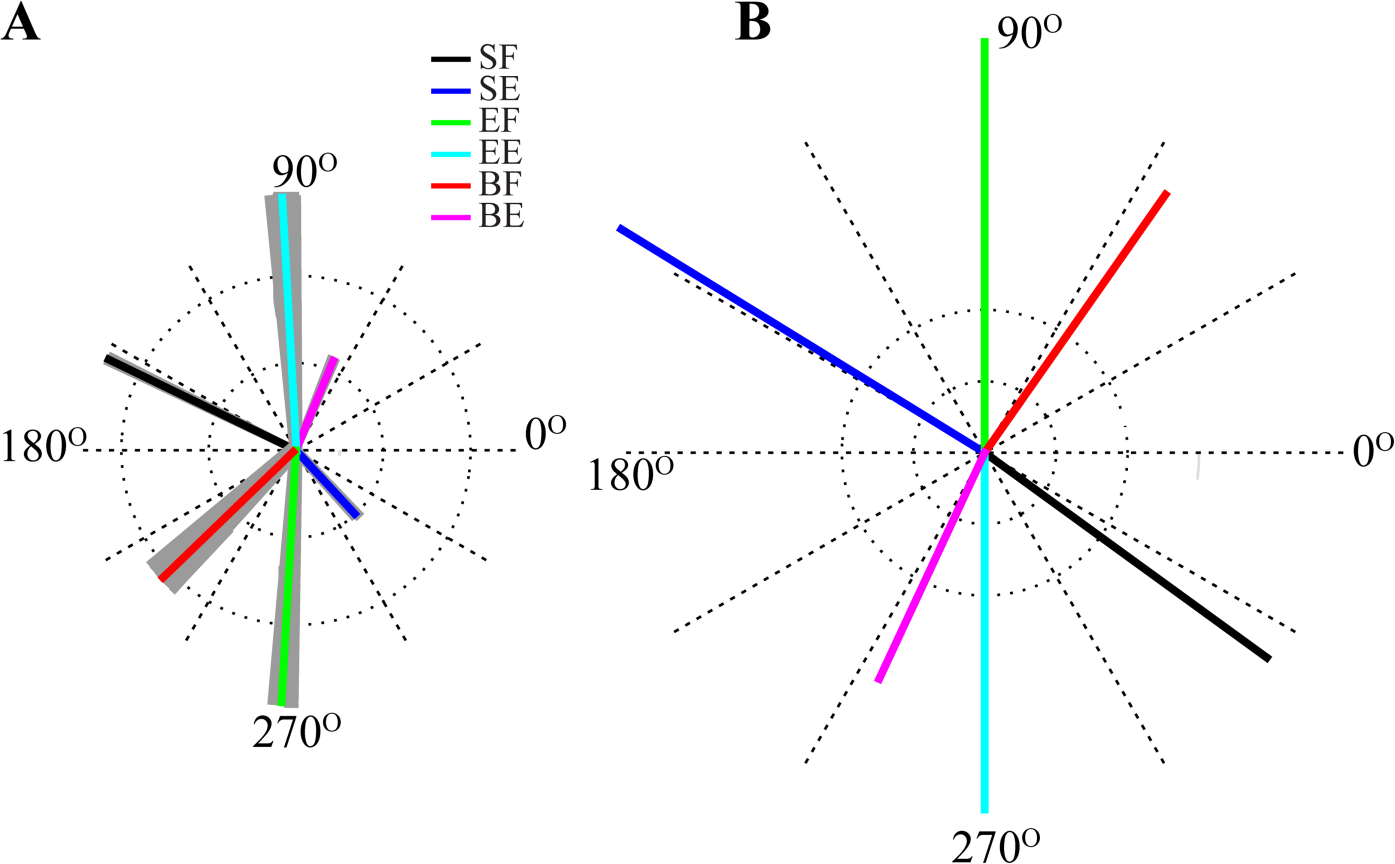
Distribution of preferred directions. Preferred directions (PDs) of cortical neurons (**A**) and Ia afferents (**B**) are fairly uniformly distributed over 360º. Colored lines show averaged PDs of cortical neurons and Ia afferents (FBIa) for corresponding muscles. The gray areas around each line represent standard deviation across 50 simulations with randomly chosen torque distribution parameter values. The length of each vector reflects the index of directional modulation. PDs of antagonist flexor and extensor related neurons and feedback are 180º apart. PDs of cortical activity are similar to the PDs of the antagonist Ia feedback. Please note that the afferents are independent of the torque distribution and have the same PD for all 50 simulations.

The analysis of activity of both spinal motoneurons and Ia afferents showed directional tuning properties similar to M1 neurons (see Fig 5). However, the correlation coefficients between neuronal activity and PDs show that spinal motoneurons have weaker directional modulation compared to M1 neurons. Ia afferent feedback also demonstrates directional modulation which have more accurate fits with cosine tuning curves compared to neuronal activity **(**Fig 5B**).** The PDs of Ia afferent feedback was fairly uniformly distributed over 0^o^ ~ 360^o^ (Fig 6B) similarly to distribution of M1 cortex cell PDs recorded by Fu et al. (see Fig 2 in [13]). Moreover, the PDs of Ia afferents were opposite to the PDs of their corresponding M1 neurons (Fig 6A and 6B).

In the model, directional modulation of the Ia afferent feedback signals emerges from their explicit dependence on muscle lengths and velocities (see Eq (5)). This is consistent with the results of Jones et al. [35] who showed that directional tuning of individual muscle afferents during voluntary wrist movements in humans can be predicted from the length changes of the corresponding muscle.

To quantify the effects of feedback on cortical activity, we increased the amplitude of feedback signals to spinal motoneurons 2-, 5- and 10-fold. The outcome showed that as the amplitude of feedback signals increased, cortical activity became more directionally tuned to a PD opposite to the corresponding feedback signal’s PD. For example, when feedback inputs were amplified by 2, 5 and 10-fold, the coefficient of determination for the directional tuning curve of SE-M1 activity increased from 0.47 to 0.69, 0.85, and 0.9, respectively. Moreover, removing the feedback signals decreases the coefficient of determination to 0.1. These results support the idea that the cortical activity may be modulated by afferent feedback signals. Obviously, amplification of the feedback signals does not affect the directional tuning curves of the spinal motoneurons.

**Fig 7:**
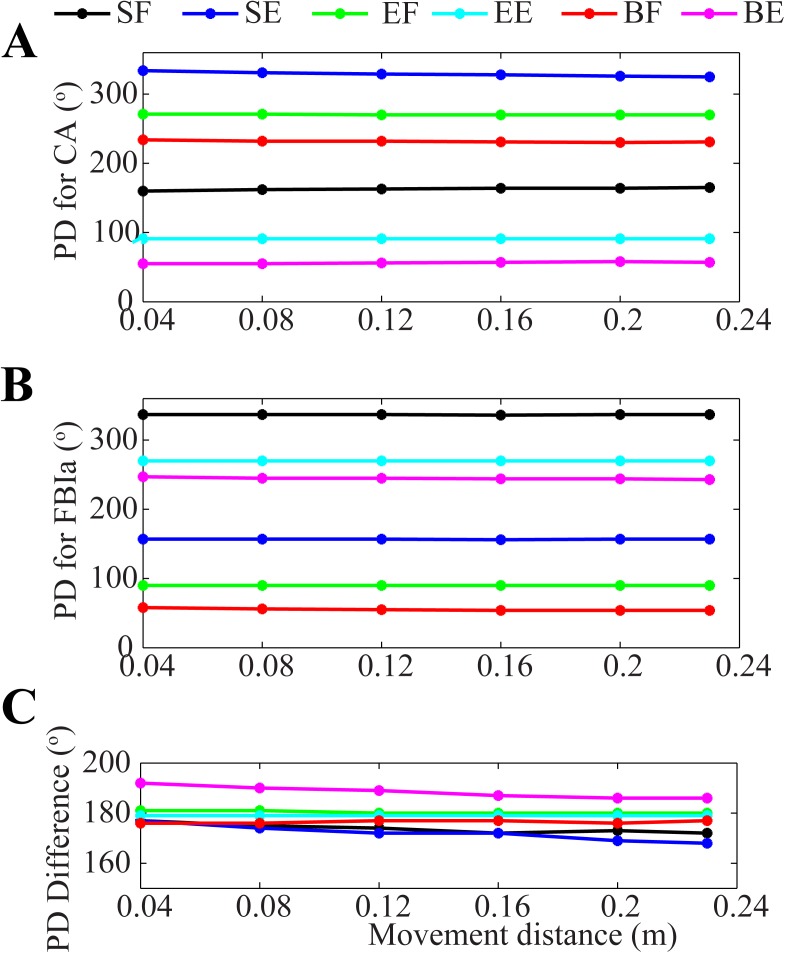
Preferred directions are invariant of reaching distance. The PDs of cortical neurons (CA) **(A)** and Ia feedback (FBIa) **(B)** are independent of reaching movement distance, which is in agreement with experimental results of Fu, Suarez et al. [13] (compare with Fig 3A in [13] showing that the PDs of 17 recorded cells over varying reaching distances are preserved). **(C)** The difference between the PD of each population and that of its corresponding Ia feedback is about 180º over six different reaching distances.

To examine the effect of reaching distance on directional modulation of cortical and feedback activity, we tested 6 different reaching distances and observed no significant change in the directional tuning curves of M1 neurons and Ia afferents. The PDs of M1 neurons and Ia afferent feedback remained relatively constant over the 6 reaching distances used (Fig 7), suggesting that PD is independent of reaching distance. Similarly, Fu, Suarez et al. [13] showed that PDs of neurons in the premotor and primary motor cortices of monkeys were independent of reaching distance (see Fig 3A in [13]). The preservation of PD across different reaching distances and speeds was also observed by Churchland, Santhanam et al. [36]. Moreover, the relationship between PDs of M1 neurons and Ia afferents was also independent of reaching distance; the difference between PDs of M1 neurons and their corresponding Ia afferents remained approximately 180^o^ over all 6 reaching distances (Fig 7C).

### Directional modulation of the contractile components (force-length and force-velocity) in the muscle force

**Fig 8:**
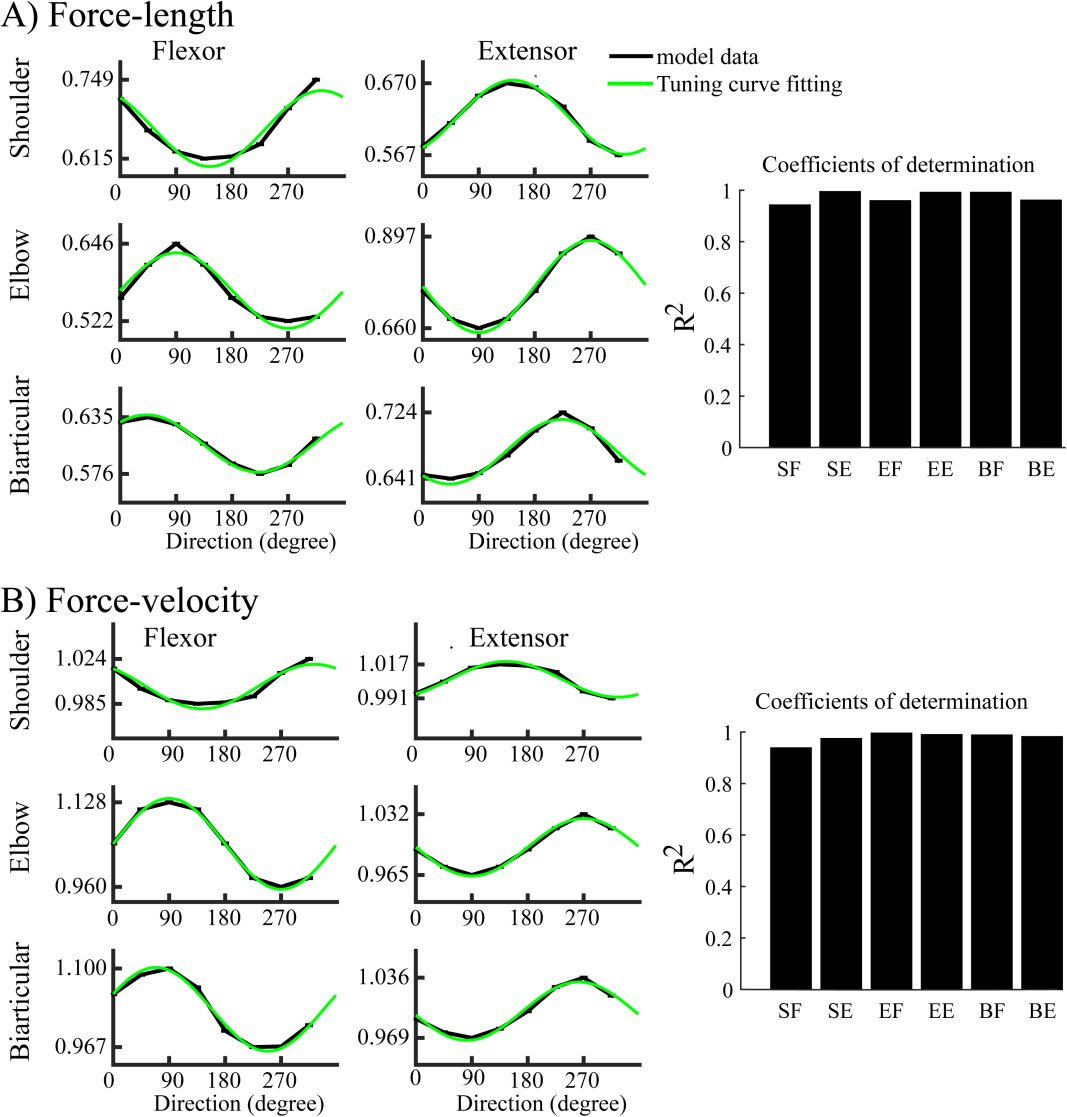
Directional modulation of contractile components of the muscle force. Dependence of force-length (A) and force-velocity (B) components of muscle force for six arm muscles on the movement direction. All profiles precisely follow cosine tuning curves shown in green (*R*^2^ is close to 1 for each muscle; see right column of the figure).

While reaching movements are performed in different directions, six arm muscles in our model contracted (shortened) or stretched (lengthened) with different magnitudes depending on the direction of the movement. Fig 8 shows the directional modulation of the muscle force. The averaged force-length (Fig 8A) component of each muscle was highest for a distinct (preferred) direction, and lowest for the opposite (anti-preferred) direction, with a smooth transition in between. Cosine functions provided excellent fits (R^2^ > 0.95, see Fig 8A, green curves) for the force-length components of all six muscle. The contractile components of the antagonist muscles demonstrated opposite PDs and tuning curves (Fig 8A), consistent with the findings of Cherian A. et al. [37], who showed that EMG activity of biceps and triceps had opposite directional tuning.

Similar to force-length components, the averaged force-velocity component of each muscle strongly correlated with the movement direction in our simulations. Specifically, it exhibited the same PD as that of the averaged force-length component, and had a similar tuning curve (Fig 8B).

### Directional modulation of other movement variables

**Fig 9:**
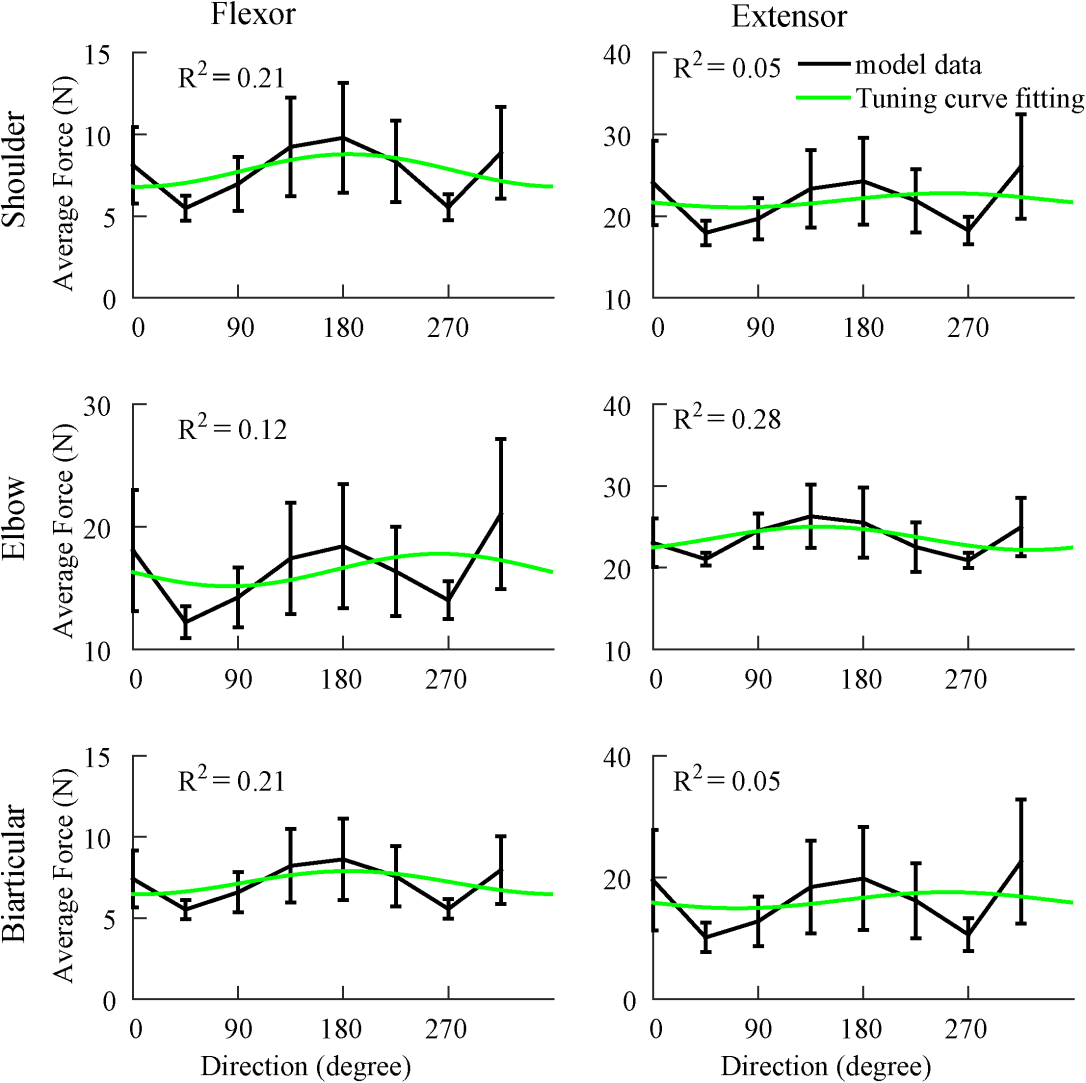
Muscle forces do not have significant directional dependence. Average forces for the six arm muscles (black) fitted with cosine tuning curves (green). The R^2^ is relatively small, implying that muscle forces are not directionally tuned. Error bars show standard deviations across 50 trails with randomly chosen motor strategy.

An identical velocity profile, with fixed peak velocity, was used to model the arm’s movements in all directions. Accordingly, the magnitude of the acceleration of the arm endpoint was not dependent on the direction; neither were the magnitudes of the joint torques (which primarily depend on joint angular accelerations). To determine if muscle forces had any correlation with movement direction, we performed 50 simulations where the torque distribution parameter *d* was varied from trial to trial in the range (0.5 ~ 1) using a uniform probability distribution. The results demonstrated that muscle forces were very weakly directionally tuned compared to cortical input tuning, muscle contractile component tuning, and feedback tuning (Fig 9); for all six arm muscles, the correlation coefficient between force and movement direction was relatively small (0.05 to 0.4). Muscle forces had large standard deviations (2.5 to 10 N), and although average muscle forces slightly changed with direction, the change was not significant. We further checked that weak correlations shown in Fig 9 emerge from the effects of joint viscosities (frictional forces, see Methods), as eliminating viscose friction forces in the joints decreased the six muscle forces’ correlations to almost zero (not shown). In summary, muscle forces are weakly modulated by the direction of the reaching movement compared to the modulation of spinal motoneurons and cortical neurons.

### Shoulder-centered reference frame

Although Cartesian coordinates have been frequently used as a reference frame for center-out reaching tasks, coordinate systems connected to the joints (shoulder or elbow) can also be used to represent the direction of the arm movement. It was previously suggested that cortical neurons, both in the motor and premotor areas, encode movement directions with an intrinsic coordinate system representing the shoulder-centered reference frame [38, 39].

**Fig 10:**
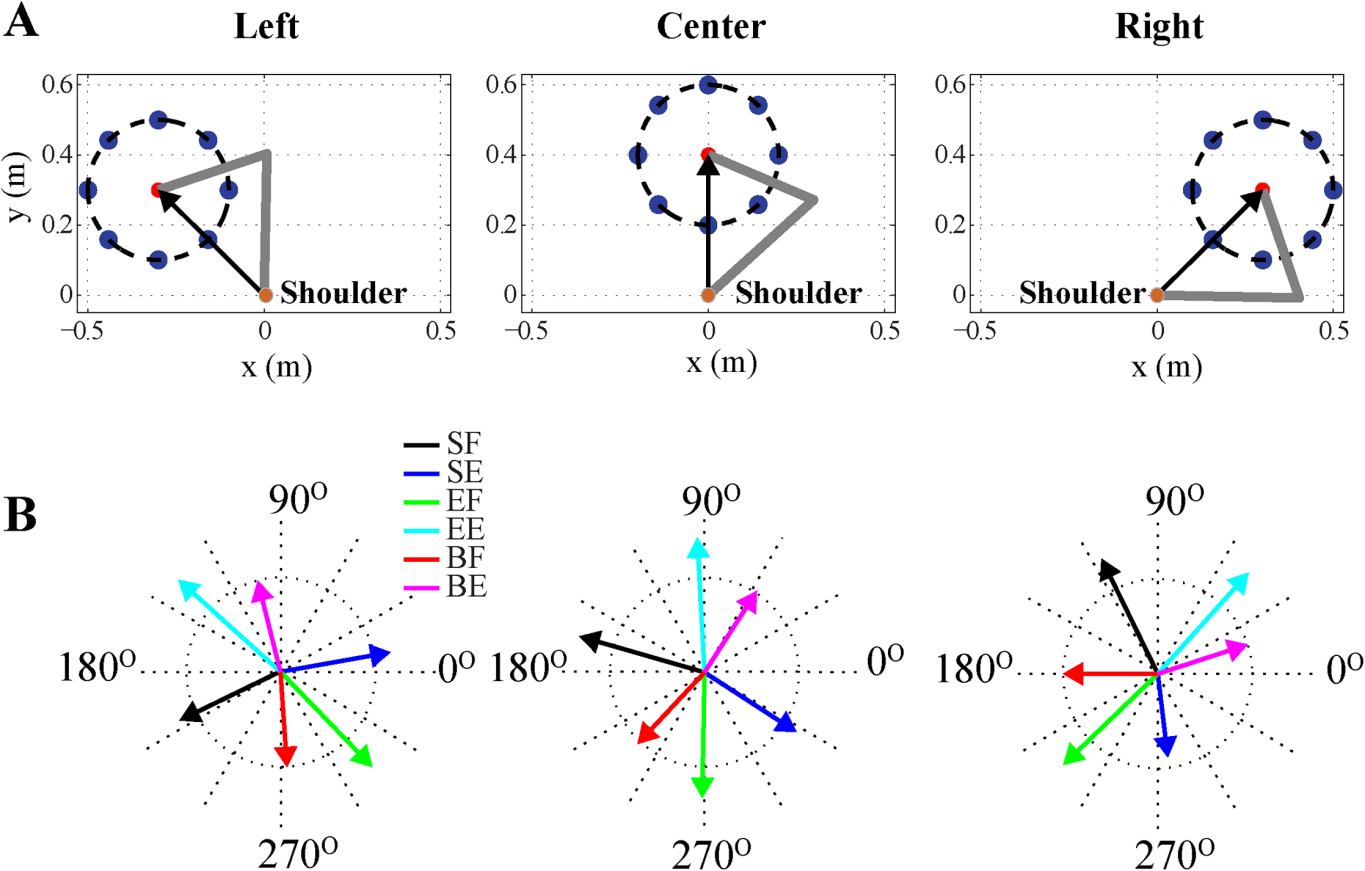
Rotating the workspace by an angle shifts the preferred directions in the same direction by the same angle. **(A)** Rotation of the workspace about the shoulder. The default workspace (center) was rotated by 45º counter-clockwise (Left), or by 45º clockwise (Right). **(B)** The distribution of preferred directions for the three workspaces corresponding to panel **A**. Vector lengths represent directional modulation index (see text for details).

Moving the workspace without changing the shoulder location can potentially affect the PDs and tuning curves of cortical neurons because of the changes in arm posture. To investigate this, we shifted the workspace in our model to the left and right, using the shoulder joint as a reference frame (Fig 10A). The whole workspace (including initial position and 8 targets) was translated with respect to the shoulder joint. The vector from the shoulder joint to the initial position (Fig 10A, black vector) was used as a reference for this transformation. The default workspace, used in most of our simulations, had coordinates (0.0, 0.4) for its initial position, and the vector from the shoulder joint to this initial position was parallel to the positive y-axis (Fig 10A, Center). Fig 10B (Center) shows the PD distribution of M1 neurons for the default workspace. When the workspace was rotated counter-clockwise by 45^o^ with respect to the shoulder joint (Fig 10A, Left), all preferred directions were shifted in the same direction by approximately the same angle (44.83^o^ ± 2.04^o^) (Fig 10B, Left). Similarly, when the workspace was rotated clockwise by 45^o^ with respect to the shoulder joint (Fig 10B, Right), all PDs were shifted in the same direction by approximately the same angle (45.67^o^ ± 3.8^o^) (Fig 10B, Right). The PD shift of the EF and EE neurons was exactly 45^o^ for both clockwise and counter-clockwise workspace transformations. Transforming the workspace by 20^o^ clockwise or counter-clockwise also shifted the PDs by 20^o^ clockwise or counter-clockwise (not shown). In other words, a shift in the reference frame resulted in a similar shift in the PDs of M1 neurons, which means that the relationship between movement direction and activity of M1 neurons is invariant once the direction is defined relative to the line connecting the shoulder and the initial point of the movement.

In summary, rotating the workspace about the shoulder joint shifts all PDs of M1 neurons by the same angle in the same direction. Similarly, Lillicrap and Scott [32] also showed that preferred directions shifted to the left or to the right when the work space was shifted to the left or right, respectively (see Fig 6 in their paper). The changes in muscle lengths during the movement largely depend on the relative direction to the shoulder joint because of the rotational symmetry of the arm geometry. Hence, in our model the directional modulation manifests as dependence on the angle between the direction of movement and the direction from the initial position to the shoulder joint. This is consistent with an idea that the motor cortex encodes direction based on the shoulder reference frame, suggested in other experimental [38, 39] and modeling [33] studies.

### Directional modulation and the movement distance

**Fig 11:**
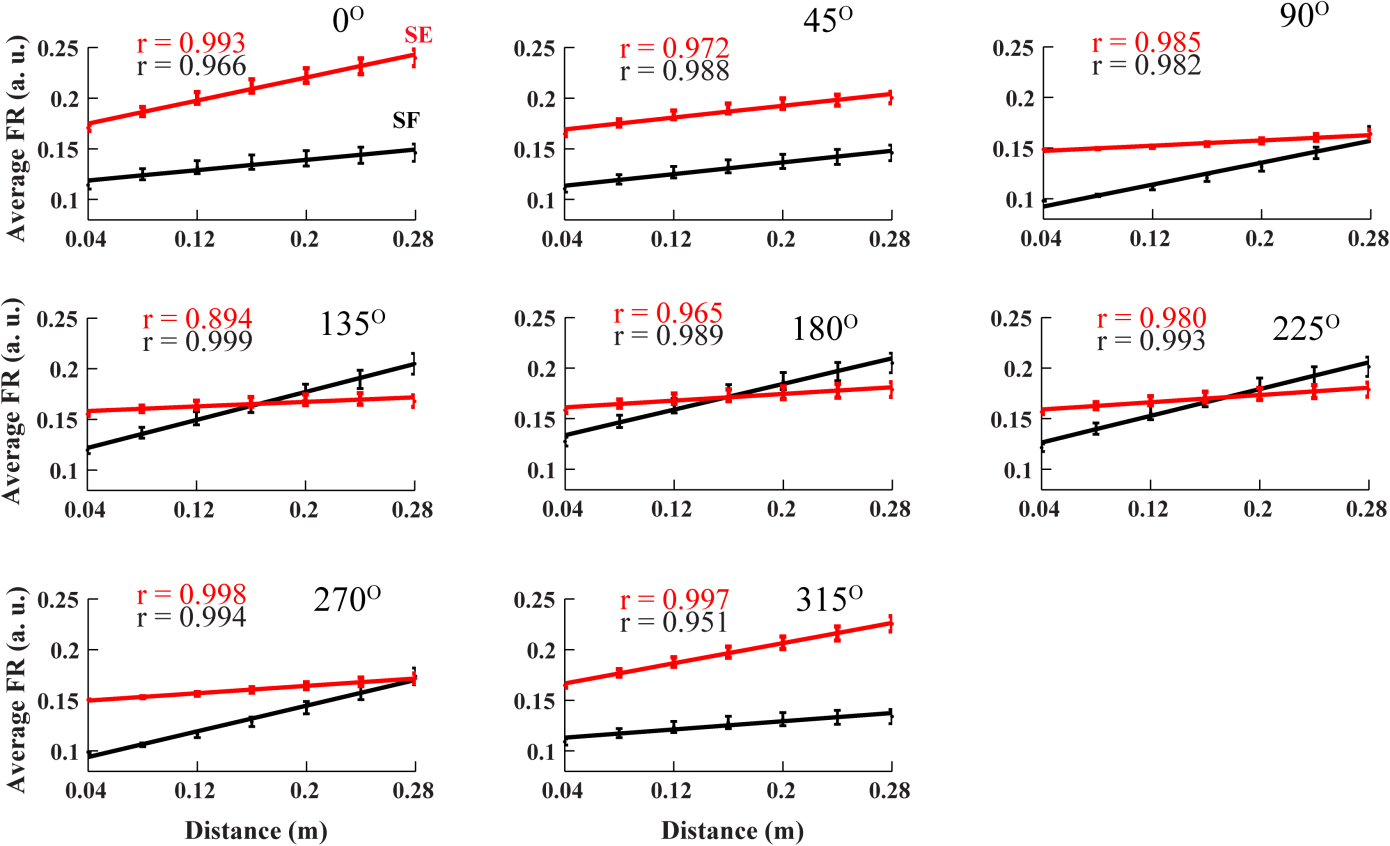
Cortical activity strongly correlates with reaching distance. The average cortical activity of the shoulder flexor (black traces) and the shoulder extensor (red traces) related neurons linearly increase with the reaching distance for the center-out task in all directions when the reaching time is fixed (1 second). Subplots show representative simulations made in 8 directions at 45° intervals. Error bars are the standard deviations across trials with randomly chosen motor strategy.

To evaluate the relationship between cortical activity and reaching distance, we simulated center-out reaching tasks in 8 directions with 7 reaching distances per direction. Since the reaching time was fixed based on previous experimental studies [13, 40], both the peak velocity and peak acceleration increased as the reaching distance increased. In our model, the average activity of each M1 neuron increased monotonically with the reaching distance (Fig 11). However, the changes in average firing rate with respect to the distance were different across M1 neurons, and depended on the movement direction as well. Our simulation results are consistent with experimental findings of Fu et al. [13] who also showed a direction dependent increase in the average firing rate of cortical neuron with increasing reach distance (see Fig 4 in [13]).

Due to constant reach time, greater reaching distances result in higher peak acceleration of the arm endpoint. In our model, the dependence of cortical activity on the reaching distance emerges from strong correlation of the latter with peak acceleration. Greater acceleration requires stronger torques at the joints, and hence, stronger activation of M1 neurons. To evaluate this hypothesis experimentally, it would be sufficient to vary the reaching time in proportion to reaching distance, such that longer reaching distances require a greater reaching time. In this case, the peak velocity would be fixed, and contrary to the task presented here, the peak acceleration would decrease as the distance increases. In our model, such a change results in the decline of average cortical activity with increased reaching distance (not shown). We suggest that correlation of cortical neuronal activity with reaching distance may depend on experimental constraints, e.g. reaching time limitations. Hence, the correlation of M1 neurons with distance emerges from the correlation between M1neurons and peak velocity or peak acceleration.

## Discussion

Most motor cortical neurons were found to have specific preferred directions in which their mean firing rate during reaching movement was maximal [2, 13]. Some studies showed that the activity of cortical neurons correlated with torques at joints and muscle tensions [41, 42], or with other kinematic variables [13, 19, 43]. It has also been suggested that the activity of a particular cortical neuron may correlate with more than one movement parameter [13, 14, 18, 44, 45]. Although many previous studies addressed these phenomena (see for review [9, 46–48]), the origin of the directional tuning properties continues to be subject to debate. Neuronal activity in the motor cortex may reflect the complexity of movement dynamics [49] as well as higher-order features such as cognitive information related to a motor task or function [50]. This complexity has led to different viewpoints and interpretations [51].

During center-out reaching movements, average values of muscle forces and joint torques do not have pronounced directional dependence, so that anisotropy of motor cortical activity is truly non-trivial. The model presented here suggests that directional tuning in the primary motor cortex is the result of movement geometry and muscle dynamics. Arm muscles contract and stretch during reaching. Geometrically, the magnitude of muscle contraction or stretching depends on movement directions. As a result, muscle state variables such as muscle lengths and their rate of change (muscle velocities) have significantly different average values when the movement is performed in different directions. Our simulations have demonstrated that averages of muscle state variables fit perfectly the cosine tuning curves. This leads to similar directional tuning of afferent feedback and muscle contractile components, which are functions of muscle state variables.

### Mechanisms of directional modulation

Most studies agree that M1 neurons are sensitive to movement directions, but fail to explain how directionally tuned motor commands produce non-directionally tuned endpoint kinematics (arm endpoint acceleration and velocity) and kinetics (net joint torques), or why non-directional endpoint variables require directionally tuned motor commands.

**Fig 12:**
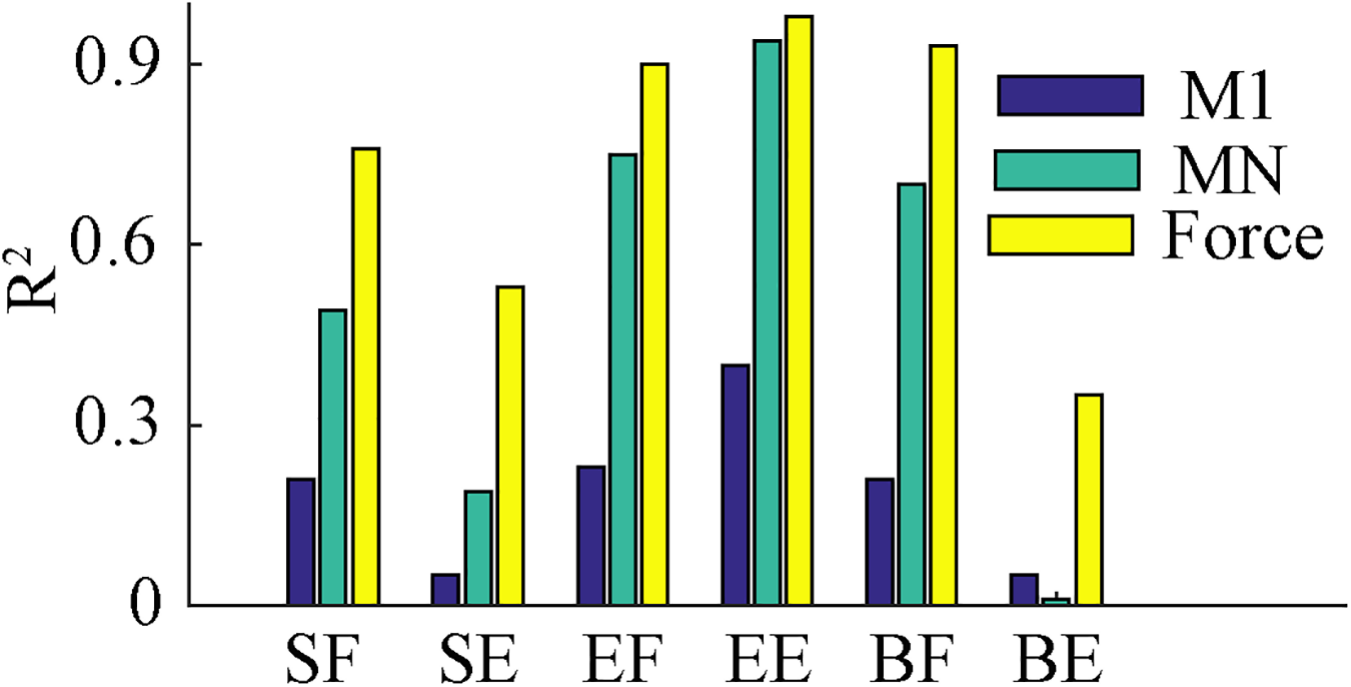
Directional modulation becomes more pronounced from muscle forces through motoneuron activity to cortical level. Coefficients of determination for cosine fits of directional dependence of time-averaged muscle forces (blue), motoneuronal outputs (cyan), and cortical inputs (yellow). Muscle forces are less directionally modulated than spinal motoneurons, which in turn are less directionally tuned than M1 neurons.

Different factors contribute to directional modulation at different levels of the system’s hierarchy. At the lowest level, frictional forces are proportional to joint angular velocities. The latter are controlled by muscle stretch/contraction and, hence, have directional modulation similar to muscle velocities. Therefore, since muscle forces have components working against frictional forces, they should exhibit some directional modulation. However, the resulting correlation between muscle forces and movement direction is pretty weak (Fig 12, blue bars) due to relatively weak friction in the joints. As an obvious implication, one can expect more pronounced directional modulation of muscle forces at higher viscous friction, e.g. if reaching movements are performed under water.

At the next level, we found that motoneurons showed stronger directional modulation than muscle forces (Fig 12, green bars). Muscle forces induced by inputs from motoneurons depend on muscle lengths and velocities (see Eq (4) in Methods). This implies that for muscle forces to be directionally indifferent, motoneuronal activities should have directional modulation opposite to those of contractile components of the corresponding muscles (see Eq (15) in Methods).

Further, motoneurons are influenced by afferent feedback from the muscles arriving at the spinal cord neural network. This feedback carries information about muscle lengths and velocities, which are strongly directionally dependent. That is, supra-spinal cortical signals have to be directionally tuned in the directions opposite to those of their corresponding feedback to provide appropriate activation patterns of spinal motoneurons.

Interestingly, all factors described above are synergistic in a sense that all of them–frictional forces, muscle contractility, and afferent feedback–have the same directional modulations as the corresponding muscle lengths and velocities. This explains why directional tuning becomes more and more pronounced when ascending on the schematic in Fig 1A from net joint torques through muscle forces and motoneurons to the cortex (Fig 12).

Previous studies have also suggested that directional tuning in the motor cortex can be attributed to muscle-state variables [30], muscle-state-dependent variables [31, 52], or limb biomechanics [27, 32]. The hypothesis that directional tuning is the result of directional dependence of muscle length dynamics is not new or unique. However, our model provides additional supporting evidence and reveals several remarkable differences from previously published studies. Mussa-Ivaldi [30] described cortical activity as a linear function of muscle velocity, and suggested that directional tuning of cortical activity emerges from muscle state variables. Our results support this idea in general, but disagree with the notion that activity in the primary motor cortex is a simple linear function of the muscle velocity. Moreover, the linear relationship suggested by Mussa-Ivaldi [30], does not clearly determine whether cortical activity and muscle velocity have the same directional tuning properties or opposite ones. Our findings specifically show that motor cortical neurons and their corresponding muscle state variables, velocity and length, have opposite directional modulation. Moreover, this modulation nonlinearly propagates across the spinal cord network and affects all spinal neurons (see Fig 12).

Todorov, using a simplified mathematical model [31, 52], demonstrated that activity in the primary motor cortex encodes arm muscle activity. He argued that correlation between cortical activity and high-level parameters, such as movement direction, speed, and target position, was a secondary effect. He suggested that cortical neurons are directionally tuned as a result of directional tuning of muscle state-dependent variables (forces generated due to muscle length and velocity). Similarly, contractile components in our model (force-length and force-velocity) contribute to the directional tuning of motoneurons and, thus, cortical neurons, which is in agreement with Todorov’s findings. In addition, our model illustrates the interplay between cortical signals and afferent feedback, which leads to augmented directional tuning of neuronal activity in the motor cortex compared to the spinal cord. In contrast to Todorov’s assumption that the spinal cord circuitry was not involved in directional tuning [31, 52], our model provides evidence suggesting that spinal circuitry may play a significant or even major role.

The arguments above also explain why directional modulation of cortical neuron activity is independent of the biomechanical redundancy of the arm, which we tested by simulating reaching tasks with randomly distributing the torques between mono- and bi-articulate muscles. Indeed, additional directional modulation emerging at each level is solely defined by variable dependent on muscle lengths and/or velocities, i.e. by the kinematics of movement in a particular direction. The same reasoning explains the results of Lillicrap and Scott [32], who used an optimized artificial neuron network to show that limb geometry, intersegmental dynamics, and force-length/velocity properties of muscles are dominant factors in the emergence of directional modulation.

In light of the above, the directional modulation in the cortical activity can be thought as a product of motor learning which has interesting implications for the emerging neuro-prosthetic technology, a.k.a. brain machine interface (BMI). BMI utilizes neural signals of motor area in the brain to control external devices such as a robotic arm. Even though BMI decoders attempt to use natural neuronal tuning to actuate the movement, the initial motor performance is usually poor, but improves with training engaging the same neuroplastic mechanisms that are involved in motor learning. Since proprioceptive feedback responsible for the initial directional tuning is no longer relevant for the artificial actuator, one could predict that the neuronal tuning should change during the learning process to eventually reflect the BMI decoder properties. This prediction finds substantial experimental support and may explain difficulties in interpretation of neuronal tuning during BMI control (see [53] for review).

### Directional modulation is invariant in the “right” coordinate system

We showed that transforming the workspace shifts the tuning curves (PDs) of M1 neurons and other directionally tuned variables. Similar changes in tuning properties have been observed in previous experimental studies. When the workspace was transformed counterclockwise from the left side to the middle and then to the right side of the animal’s body, the PDs of cortical neurons shifted clockwise [38]. Tanaka and Sejnowski also reported shifts of PDs when the workspace was transformed [33]. Lillicrap and Scott [32] also showed that optimal preference movement distributions change as a function of limb posture and musculoskeletal organization. In addition, it was observed that directional tuning of motor cortical neurons was affected by the posture and orientation of the monkey’s arm [23] even when the workspace remained fixed. Our analysis suggests that directional modulation remains virtually invariant with respect to the transformations above if defined in terms of the angle between reaching direction and the line connecting the shoulder joint and the initial arm position.

### Directional tuning may vary depending on task conditions

It was proposed that directional tuning in the motor cortex, as defined by Georgopulos, is not robust; it varies depending on task conditions [54, 55]. In particular, some studies showed that directional tuning changes with the limb’s velocity or reaching distance [36, 49]. Similarly, our simulations suggest that the fit of cortical activity by a cosine tuning curve gets less accurate when the movement speed is increased (not shown) which may result in significantly different (more variable) PD estimates. Neuronal activity patterns both in the cortex and the spinal cord have two components–the dynamic component aimed at generating a specific movement, and the compensatory component that counteracts the effects of directional modulation of muscle length and velocity dependent variables (frictional forces, contractile components, and afferent feedback). The latter is responsible of the directional modulation of neuronal activity. With increasing speed and/or distance of the movement, the dynamic component begins prevailing the compensatory component, and, thus, partially destroys cosine-like fits.

### Higher-level and supra-spinal structures

The spinal cord circuitry we described in this study is the low-level circuit close to output motoneurons. In our model, we assumed that, M1 neurons directly control spinal motoneurons to activate muscles in the arm. Some pyramidal neurons in M1 of monkeys do directly project to motoneurons in the spinal cord [56–58]. However, a majority of M1 neurons indirectly control them through interneurons [59]. Various types of these interneurons with complex interconnections may be involved in relaying motor commands from the motor cortex to the spinal motoneurons. The Modularity Theory, for example, suggests that all motor behaviors may be constructed by combining motor primitives generated by composite motor modules in the spinal cord [60–65]. According to this theory, the brain does not control individual muscles directly, but rather activates sets of muscles via motor primitives to produce complex movements. Therefore, directional properties of the cortical neurons controlling motor primitives would depend on geometry of multiple muscles, and our finding that cortical neurons have directional preferences opposite to the corresponding muscle lengths and velocities may not be entirely valid. However, our main conclusion about directional dependence of the neuronal activity in the higher-level and supra- spinal structures due to directional dependence of the muscle geometry still holds.

### Comparison with other models

Several previous models of arm reaching movements were developed to understand activity patterns in the motor control system and their relationships with movement parameters. In some studies, simple mathematical equations have been suggested to describe relationships between motor cortex activity and movement characteristics [30, 66]. These models, however, were synthesized to illustrate particular directional tuning hypotheses, and did not have specific biological justification.

Activity patterns of motor cortical neurons have also been modeled based on endpoint velocity, acceleration and limb position to understand tuning properties derived from muscle-state dependent variables [31, 52]. However, spinal cord circuits and biomechanics of the arm were not considered in these studies. Tanaka and Sejnowski [33], for example, developed a three-joint arm model performing reaching movements in 2D space, where muscle forces were described as a rectified sum of motor cortical activities taking into account neither muscle contractile components nor spinal reflexes. Lillicrap and Scott [32] developed a two-joint arm model controlled by six muscles for reaching movements in 2D space, which is very similar to our arm’s model. However, there are major differences between the model structures. For example, their model did not include any spinal circuits, but included complex cortical neural computations to adjust parameters in the cortical network via a learning procedure using the feedback from the arm dynamics. We can argue that this learning procedure is aimed at finding a particular solution of the same inverse problem we are addressing in our study. However, the cost function used and a specific implementation of the learning process define which particular solution is selected. That may potentially induce the directional preference of its own. In our study, we analyzed a family of possible motor programs for the movements in each direction, and confirmed that all members of each family possess similar directional properties.

In summary, Lillicrap’s and other models used optimal control theory based on specific assumptions that cortical activity is optimized to achieve certain behavioral objectives [32, 34, 67]. We do not use any assumptions about optimality. Instead, we focus on a particular type of movement, i.e. goal-directed reaching, explicitly assimilating information that reaching movements follow straight-line trajectories with a bell-shaped velocity profile as it was shown in previous experimental studies [36, 68, 69].

## Conclusion

In the present study, we proposed mechanistic explanation of the fact that during planar reaching arm movements directional dependence of time-averaged activity of cortical neurons originates from the anisotropy of average muscle lengths and velocities. Specifically, cortical neurons have opposite preferred directions compared to the “preferred” directions of lengths and velocities of corresponding arm muscles. This anti-correlation builds up as one ascends along the hierarchy of the motor control system. First, the directional preference emerges at the level of muscle forces as a consequence of viscous friction in the joints. Second, it augments at the level of spinal motoneurons to compensate for the muscle contractile component (which are functions of the muscle lengths and velocities) directional dependence. Third, neuronal directional modulation is further amplified at the cortical level to counteract the directional dependence of afferent feedback inputs carrying information about muscle lengths and velocities to the spinal cord network. To conclude, we showed that the motoneurons and afferent feedback have significant impact to modulate the directional tuning behavior of cortical neurons.

## Methods

### Biomechanical model of the arm

The Arm (see Fig 1C) is modeled as a 2-joints mechanical system of rigid segments connected by revolute joints, which operates in the horizontal plane. The dynamics of the arm’s motion are derived from the Lagrange equations and include angular velocities and accelerations, Coriolis and centrifugal forces, muscle forces, and viscoelastic forces at the joints. The parameters of mechanical system such as masses and lengths of correspondent arm segments are based on human biomechanics [70]. Kinematics of the segments is described by the following differential equations:

$$\begin{array}{l} q_{1}=I_{1}\ddot{\theta_{1}}+m_{2}L_{1}^{2}\ddot{\theta_{1}}+m_{2}L_{1}d_{2}\ddot{\theta_{2}}\cos\left(\theta_{1}-\theta_{2}\right)-m_{2}L_{1}d_{2}\dot{\theta_{2}}\left(\dot{\theta_{1}}-\dot{\theta_{2}}\right)\sin\left(\theta_{1}-\theta_{2}\right)+m_{2}L_{1}d_{2}\dot{\theta_{1}}\dot{\theta_{2}}\sin\left(\theta_{1}-\theta_{2}\right)\\ q_{2}=I_{2}\ddot{\theta_{2}}+m_{2}d_{2}^{2}\ddot{\theta_{2}}+m_{2}L_{1}d_{2}\ddot{\theta_{1}}\cos\left(\theta_{1}-\theta_{2}\right)-m_{2}L_{1}d_{2}\dot{\theta_{1}}\left(\dot{\theta_{1}}-\dot{\theta_{2}}\right)\sin\left(\theta_{1}-\theta_{2}\right)-m_{2}L_{1}d_{2}\dot{\theta_{1}}\dot{\theta_{2}}\sin\left(\theta_{1}-\theta_{2}\right) \end{array}$$ (1)

where: *q*_1_ = *q*_1*v*_ − *q*_2*v*_ + *q*_1*M*_ and *q*_2_ = *q*_2*v*_ + *q*_2*M*_ are generalized forces (torques), which include joint friction forces (*q*_1*v*_, *q*_2*v*_) and torques created by muscles (*q*_1*M*_, *q*_2*M*_); *θ*_1_, *θ*_2_ are the generalized coordinates (the angles between positive y-axis and the upper arm or forearm, respectively); $I_{1}=m_{1}L_{1}^{2}/3$ is the moment of inertia of the upper segment around an axis through the point of suspension; $I_{2}=m_{2}L_{2}^{2}/12$ is the moment of inertia of the lower segment about an axis through its center of mass; *m*_1_ = 1.79 kg, *m*_2_ = 1.55 kg are the masses of the upper and lower segments, respectively; *L*_1_ = 0.34 m is the length of the upper segment, *L*_2_ = 0.31 m is the length of the lower segment; d_1_ = *L*_1_/2 is the distance from the shoulder joint to the center of mass of the upper arm, *d*_2_ = *L*_2_/2 is the distance from the elbow joint to the center of mass of the forearm.

After simplifying Eq (1), the movement of the 2-joint arm can be described by angular accelerations at the shoulder and elbow joints given by:

$$\begin{array}{l} \ddot{\theta_{1}}=\left(a_{1}\left(q_{1}+f_{1c}\right)-b\left(q_{2}+f_{2c}\right)\right)/\left(a_{1}a_{2}-b^{2}\right)\\ \ddot{\theta_{2}}=\left(a_{2}\left(q_{2}+f_{2c}\right)-b\left(q_{1}+f_{1c}\right)\right)/\left(a_{1}a_{2}-b^{2}\right) \end{array}$$ (2)

where: $a_{1}=I_{1}+m_{2}L_{1}^{2}$, ${a_{2}=I}_{2}+m_{2}d_{2}^{2}$, ${b=m}_{2}L_{1}d_{2}\;\cos\left(\theta_{1}-\theta_{2}\right)$, $f_{1c}=m_{2}L_{1}d_{2}{\dot{\theta }}_{2}^{2}\;\sin\left(\theta_{1}-\theta_{2}\right)$, $f_{2c}=m_{2}L_{1}d_{2}{\dot{\theta }}_{1}^{2}\;\sin\left(\theta_{1}-\theta_{2}\right)$. The joint viscous friction forces are given by: $q_{1v}=-\eta_{v}\dot{\theta_{1}}$; $q_{2v}=-\eta_{v}(\dot{\theta_{2}}-\dot{\theta_{1}})$, where the viscosity parameter *η*_*v*_ = 0.05.

### Muscles

We considered six muscles, which control arm movements in horizontal plane. Specifically, four 1-joint muscles (shoulder flexor (SF), shoulder extensor (SE), elbow flexor (EF) and elbow extensor (EE)) and two 2-joints muscles (shoulder and elbow extensor (BE) and shoulder and elbow flexor (BF)) control the arm’s movements. Muscles, which perform the similar action and have similar anatomy, were substituted by one muscle with averaged parameters.

Total muscle torques, *q*_1*M*_ and *q*_2*M*_, are produced by all muscles at the shoulder and elbow and calculated as:

$$\begin{array}{l} q_{1M}=F_{SF}R_{SF}-F_{SE}R_{SE}+F_{BF}R_{BFS}-F_{BE}R_{BES}\\ q_{2M}=F_{EF}R_{EF}-F_{EE}R_{EE}+F_{BF}R_{BFE}-F_{BE}R_{BEE} \end{array}$$ (3)

where: *F* and *R* represent muscle forces and averaged moment arms [71, 72] for all muscles (see Table 1). Muscle indices are defined as follow: SF and SE represent the shoulder flexor and extensor; EF and EE represent the elbow flexor and extensor. BFS, BES represent 2-joint flexor; BES, BEE represent 2-joint extensor, respectively.

**Table 1:** Muscle model parameters.

Muscle	Maximal force, *F*_*max*_ (N)	Optimal length, *L*_*opt*_ (m)	*k*_*v*_	*V*_*max*_	Moment arm, *R* (m)
Shoulder Flexor (SF)	420	0.185	2.1	Δ∝_1_ ∙ *R*_*SF*_	0.015
Shoulder Extensor (SE)	570	0.170	2.0	Δ∝_1_ ∙ *R*_*SE*_	0.008
Elbow Flexor (EF)	1010	0.180	1.7	Δ∝_2_ ∙ *R*_*EF*_	0.035
Elbow Extensor (EE)	1880	0.055	1.7	Δ∝_2_ ∙ *R*_*EE*_	0.021
Biarticular Flexor (BF)	460	0.130	2.0	Δ∝_1_ ∙ *R*_*BFS*_ + Δ∝_2_ ∙ *R*_*BFE*_	0.020/0.036
Biarticular Extensor (BE)	630	0.150	2.1	Δ∝_1_ ∙ *R*_*BES*_ + Δ∝_2_ ∙ *R*_*BEE*_	0.005/0.021

Description of muscle forces is based on the Hill-type model proposed by Harischandra and Ekeberg [73] was also adapted to human biomechanics. The total force *F* for each muscle is calculated as:

$$F=F_{max}\cdot \left(MN\cdot F_{l}\cdot F_{v}+F_{p}\right)$$ (4)

where: *F*_*max*_ is the maximum force (see Table 1) based on experimental data [71, 72]; *MN* is the activity of the corresponding motoneuron; *F*_*l*_, *F*_*v*_ are the normalized length- (*F*_*l*_) and velocity- (*F*_*v*_) dependent variables describing the muscle contractile component and *F*_*p*_ is the passive parallel component. The length- (*F*_*l*_) and velocity- (*F*_*v*_) dependent components of contractile elements are described as: *F*_*l*_ = exp(−|(*l*^2.3^ − 1)/1.26|^1.62^) and *F*_*v*_ = (−0.69 − 0.17*v*)/(*v* − 0.69) if *v* < 0 or *F*_*v*_ = ((5.34 ∙ *l*^2^ − 8.41 ∙ *l* + 4.7) ∙ *v* + 0.18)/(*v* + 0.18) if *v* ≥ 0. Passive element (*F*_*p*_) is calculated as: *F*_*p*_ = 3.5 ∙ ln(exp((*l* − 1.4)/0.005) + 1) − 0.02 ∙ exp(−18.7 ∙ (*l* ∙ 0.79)) − 1). The muscle lengths (*l*) and velocities (*v*) for different muscles are specified in Table 2.

**Table 2:** Muscle lengths and velocities.

Muscle	Length, *l* (m)	Velocity, *v* (m/sec)
Shoulder Flexor (SF)	$L_{opt}\frac{\propto_{1}^{max}-\theta_{1}}{{∆\propto }_{1}}H(\propto_{1}^{max}-\theta_{1})$	$-\dot{\theta_{1}}R_{SF}$
Shoulder Extensor (SE)	$L_{opt}\frac{\theta_{1}-\propto_{1}^{min}}{{∆\propto }_{1}}H(\theta_{1}-\propto_{1}^{min})$	$-\dot{\theta_{1}}R_{SE}$
Elbow Flexor (EF)	$L_{opt}\frac{\propto_{2}^{max}-(\theta_{2}-\theta_{1})}{{∆\propto }_{2}}H(\propto_{2}^{max}-(\theta_{2}-\theta_{1}))$	$-(\dot{\theta_{2}}-\dot{\theta_{1}})R_{EF}$
Elbow Extensor (EE)	$L_{opt}\frac{(\theta_{2}-\theta_{1})-\propto_{2}^{min}}{{∆\propto }_{2}}H((\theta_{2}-\theta_{1})-\propto_{2}^{min})$	$-(\dot{\theta_{2}}-\dot{\theta_{1}})R_{EE}$
Biarticular Flexor (BF)	$L_{opt}\frac{\propto_{1}^{max}+\propto_{2}^{max}-\theta_{2}}{{∆\propto }_{1}+{∆\propto }_{2}}H(\propto_{1}^{max}+\propto_{2}^{max}-\theta_{2})$	$-\dot{\theta_{1}}R_{BFS}-(\dot{\theta_{2}}-\dot{\theta_{1}})R_{BFE}$
Biarticular Extensor (BE)	$L_{opt}\frac{\theta_{2}-\propto_{1}^{min}-\propto_{2}^{min}}{{∆\propto }_{1}+{∆\propto }_{2}}H(\theta_{2}-(\propto_{1}^{min}+\propto_{2}^{min}))$	$-\dot{\theta_{1}}R_{BES}-(\dot{\theta_{2}}-\dot{\theta_{1}})R_{BEE}$

*H*(.) is the Heaviside step function. Based on experimental data [74], the angle limitations at the shoulder joint are $\propto_{1}^{max}=55^{o}$ and $\propto_{1}^{min}={-135}^{o};$ the angle limitations at the elbow joint are $\propto_{2}^{max}=155^{o}$ and $\propto_{2}^{min}={-5}^{o};$ ${∆\propto }_{1}=0.97{(\propto }_{1}^{max}-\propto_{1}^{min})$; and ${∆\propto }_{2}={0.97(\propto }_{2}^{max}-\propto_{2}^{min})$ for both Tables 1 and 2.

### Afferent feedback

Muscle afferent feedback activities (*Ia* and *Ib* feedback signals) are derived and modified from the formulas suggested by Prochazka [75]:

$$\begin{array}{l} Ia=k_{v}\cdot v_{norm}^{p_{v}}+k_{dI}\cdot d_{norm}+k_{nI}\cdot y+{const}_{I}\\ \;Ib=k_{F}.F_{norm} \end{array}$$ (5)

where: *v*_*norm*_ = *v*/*V*_*max*_ is the normalized muscle velocity, *d*_*norm*_ = (*l* − 0.2 ∙ *L*_*opt*_)/*L*_*opt*_ is the normalized muscle lengthening if *l* > *L*_*opt*_ or 0 otherwise, *y* is the output activity of the corresponding motoneuron, *F*_*norm*_ = (*F* − 0.1 ∙ *F*_*max*_)/*F*_*max*_ is the normalized muscle force.

Parameters *V*_*max*_, *L*_*opt*_ and *F*_*max*_ are based on averaged parameters of correspondent muscles of upper of human male dataset [71, 72] and specified in Table 1. The coefficients *k*_*v*_ are optimized in order to normalize Ia signal from each muscle operating within locomotor range (see Table 1). The remaining coefficients are defined as follows: *k*_*dI*_ = 0.8, *k*_*nI*_ = 0.05, *k*_*F*_ = 1.0, *const*_*I*_ = 0.01.

### Spinal neuronal network

The Spinal Cord circuit provides direct control of the arm biomechanical model via corresponding motoneurons (Fig 1A). Although the activity of the motor cortex is correlated to muscle activity during voluntary movements such as reaching task [9–11], spinal reflexes still play an important role in arm kinematics [76]. In our model of the spinal cord we implemented several basic reflexes such as: stretch reflex; autogenic inhibition reflex; and recurrent inhibition of motoneurons via Renshaw cells (see Fig 1B).

A simplified scheme of the *stretch reflex* including monosynaptic excitation of synergist motoneurons and disynaptic Ia reciprocal inhibition circuitry is based on previously published work [77]. The additional Ia-interneurons, which receive Ia afferent input and mediate inhibition to antagonist motoneurons, are introduced for all pairs of antagonist muscles. These interneurons also receive mutual inhibition from antagonist Ia-interneurons.

Because of *autogenic inhibition reflexes*, activation of Golgi tendon organ Ib afferents evokes inhibition of synergist motoneurons [78, 79]. The inhibitory pathway includes an additional Ib-interneuron that receives an excitatory Ib feedback from the corresponding muscle and relays the inhibitory signal to the same motoneuron and its synergists. Schematic of this reflex is based on previously described circuitry [77, 80].

The activity of motoneurons is also regulated by Renshaw cells [81]. These interneurons innervate and inhibit the very same motoneurons which activate them as well as synergetic motoneurons [82]. Renshaw cells also inhibit ipsilateral interneurons which provide Ia reciprocal inhibition [78]. In addition, the activity of Renshaw cells is regulated by the activity of contralateral Renshaw cells excited by antagonist motoneurons (see Fig 1B).

Each neuron in our model represents the activity of a population of corresponding spiking neurons and is based on a non-spiking description of the neuron model. The output activity (firing rate) of each neuron (*y*) is represented by a sigmoidal function of the aggregate input:

y=s(v)=(1+exp{−(v−v12)/k})−1 (6)

where $v=b+\sum_{i=1}^{N}w_{i}x_{i}$; *x*_*i*_ is the *i*-th input and *w*_*i*_ is the synaptic weight (or strength of connection) between the *i*-th input and the neuron, *b* is a bias term that controls the excitability of the neuron, and *s*(*v*) is an activation function. The parameters v12=0.5 and *k* = 0.1 in Eq (6) specify the threshold and the slope of the activation function, respectively.

As mentioned above, the spinal cord neuronal network consists of six local circuits which are responsible for controlling corresponding muscles. Each circuit includes a motoneuron (MN), Renshaw cell (RC), Ia-interneuron, and Ib-interneuron. The bias term was the same (*b* = −0.28) for all neurons in the spinal cord.

The gains of the feedback inputs were chosen in such a way that all reflexes modulate the motoneuron activity within 15% of maximum. This roughly corresponds to existing experimental data on contribution of reflexes in the EMG activity during voluntary limb’s movements [83, 84]. The connections in the network are defined as follows [80] (see also Fig 1B). The synaptic weights are indicated in brackets.

- RCs receive excitation from corresponding MNs (+0.25).
- RCs inhibit antagonist RCs (-0.25).
- RCs inhibit corresponding MNs (-0.25).
- Flexor (extensor) RCs also inhibit all other flexor (extensor) MNs (-0.125).
- RCs inhibit Ia interneurons (-0.25).
- Ia interneurons inhibit antagonist Ia interneurons (-0.25).
- Ia interneurons inhibit antagonist MNs (-0.25).
- Ib interneurons inhibit corresponding MNs (-0.25).
- Flexor (extensor) Ib interneurons inhibit all other flexor (extensor) MNs (-0.125).
- Ib interneurons are excited by corresponding Ib feedback (+0.15).
- MNs and Ia interneurons are excited by corresponding Ia feedback (+0.15).
- MNs, Ia, and Ib interneurons are excited by corresponding cortical neurons (+0.15).

The spinal cord neuronal network contains recurrent connections, and, hence, the firing rates cannot be calculated in a feed-forward way. We assumed that the firing rate variables of the network are in instantaneous equilibrium, which can be found by an iterative procedure explained below. The “current” state of the network, ${\vec{Y}}_{k}$, that includes spinal motoneuron activities as well as firing rates of all spinal interneurons, is described by:

$${\vec{Y}}_{k+1}=s\left(\vec{B}+W\cdot {\vec{Y}}_{k}+\vec{MC}+\vec{FB}\right)$$ (7)

where ***W*** is the matrix of connection weights between elements (including ascending feedback, neuron outputs, and descending signals from upper levels, see above) in the network, vector $\vec{B}$ consists of bias terms for all neurons in the network, *k* is the iteration number, $\vec{MC}$ is the input from the motor cortex, $\vec{FB}$ is a vector of afferent feedback from the muscles, and *s* is the sigmoid activation function (6). Eq 7 is iterated until ${\vec{Y}}_{k}$ reaches a steady state with a preset tolerance. Its equilibrium value, $\vec{Y}$, is accepted as the network response to the cortical input $\vec{MC}$ given the feedback $\vec{FB}$. We verified that with the synaptic weights used (***W***) the map (7) always had a unique stable equilibrium. Hence, the network state can be considered as a function of the cortical inputs and afferent feedback signals:

$$\vec{Y}\left(\vec{MC},\vec{FB}\right)=\lim_{k\to \infty }{\vec{Y}}_{k}$$ (8)

calculated by iterating (7).

### The cortical controller

We model the execution of a reaching task by moving the arm endpoint (the wrist) from a given initial position to a desired target position. Based on the specified target position, the model calculates a set of activity profiles for the motor cortical neurons (a motor program), which provide the wrist movement along a defined trajectory, ending at the target position. Given the initial and target positions of the arm’s endpoint in the (*x*, *y)* orthogonal coordinate system, and preset reaching time, we first calculate muscle forces required to generate the desired motion along a straight-line trajectory with a defined velocity profile. Using the muscle forces, we then calculate required motoneuron activity (motoneuron signals), and finally supra-spinal input generated by motor cortical neurons.

### Arm trajectory and joint angles

Let the initial and target positions of the arm’s endpoint be (*x*_1_, *y*_1_) and (*x*_2_, *y*_2_), respectively. The velocity profile along the trajectory, *v(t)*, of the arm’s endpoint is defined as follows:

$$v\left(t\right)=\frac{L}{T}\left(1-\cos\left(\frac{2\pi t}{T}\right)\right),$$ (9)

where *T* is the reaching time (time to reach the target), *L* is the distance between the initial and target positions in meters (reaching distance), *v*(*t*) is in m/s and time *t* is in seconds. The velocity has a bell-shaped profile and is zero at the initial and target positions. The peak velocity is controlled by *T* and *L*; the inverse relationship between reaching time and peak velocity has been experimentally reported [85]. Using (9), we can calculate the dependence of the endpoint coordinates on time as follows:

$$\begin{array}{l} x(t)=x(0)+U_{x}\int_{0}^{t}v(t)dt=x_{1}+(x_{2}-x_{1})\left(\frac{t}{T}-\frac{1}{2\pi }\sin\left(\frac{2\pi t}{T}\right)\right),\\ y(t)=y(0)+U_{y}\int_{0}^{t}v(t)dt=y_{1}+(y_{2}-y_{1})\left(\frac{t}{T}-\frac{1}{2\pi }\sin\left(\frac{2\pi t}{T}\right)\right). \end{array}$$ (10)

Here *U*_*x*_ and *U*_*y*_ are the components of the unit vector along the trajectory. We then calculate the coordinates of the elbow joint (*x*_*e*_, *y*_*e*_) by finding the intersection of two circles with centers at the origin (shoulder) and at the endpoint of the arm with radii *L*_1_ and *L*_2_, respectively (see Fig 1C):

$$\begin{array}{l} x_{e}^{2}+y_{e}^{2}=L_{1}^{2}\\ {\left(x-x_{e}\right)}^{2}+{\left(y-y_{e}\right)}^{2}=L_{2}^{2} \end{array}$$ (11)

Given the time dependencies of the wrist and elbow coordinates, we calculate the joint angles and their first and second derivatives using these simple geometrical relationships:

$$x_{e}=L_{1}sin\;\theta_{1};y_{e}=-L_{1}cos\;\theta_{1};x-x_{e}=L_{2}sin\;\theta_{2};y-y_{e}=-L_{2}cos\;\theta_{2}$$ (12)

by differentiating Eqs (10), (11) and (12) twice with respect to time and solving the resulting equations for $\theta_{1},\theta_{2},{\dot{\theta }}_{1},{\dot{\theta }}_{2},\ddot{\theta_{1}},\ddot{\theta_{2}}$. We omit the explicit formulas due to their complexity. As a result of these procedures, we obtain the values for the joint angles and their first and second derivatives (angular velocities and angular accelerations) at every time *t* during the reaching movement.

### Muscle forces

Using Eq (2) and the angular accelerations, we calculate total torques *q*_1_ and *q*_2_ at the shoulder and elbow joints:

$$\begin{array}{c} q_{1}=a_{1}{\ddot{\Theta }}_{1}+b{\ddot{\Theta }}_{2}\\ q_{2}=a_{2}{\ddot{\Theta }}_{2}+b{\ddot{\Theta }}_{1} \end{array}$$ (13)

which allows us to calculate the torques generated by the muscles after subtracting frictional torques:

$$\begin{array}{l} q_{1,M}=q_{1}-\left(q_{1,v}-q_{2,v}-q_{2,M}\right)\\ q_{2,M}=q_{2}-q_{2,v} \end{array}$$ (14)

where *q*_1,*M*_ and *q*_2,*M*_ are the total muscle torques at the shoulder and elbow joints, respectively; *q*_1,*v*_ and *q*_2,*v*_ are the frictional torques in the shoulder and elbow joints, respectively, that are defined by angular velocities (see Eq (2) and the text below it).

Since torque values (*q*_1,*M*_, *q*_2,*M*_) are created by 6 muscles, there may be significant redundancy in possible muscle activation patterns. To limit possible solutions, we made the following assumptions: 1) positive torque values correspond to flexor activity, and negative torque values correspond to extensor activity; 2) since bi-articular muscles are concurrently active with shoulder and elbow muscles, there are infinitely many ways to distribute the load over the muscles to provide the same aggregate torque. Therefore, we introduced a dimensionless control parameter *d* which dictates how the torque at the shoulder joint is distributed between the bi-articular muscles and single flexor and extensor muscles. Thus, muscle forces were calculated using the following equations:

$$\begin{array}{c} d(F_{SF}R_{SF}+{-F}_{SE}R_{SE})+(d-1){(F}_{BF}R_{BFS}+{-F}_{BE}R_{BES})-q_{1M}=0\\ F_{EF}R_{EF}+{-F}_{EE}R_{EE}+F_{BF}R_{BFE}+{-F}_{BE}R_{BEE}-q_{2M}=0, \end{array}$$

where *d* represents torque distribution about the shoulder, and other notation are similar to those in Eq 6. We chose to include the torque distribution parameter at the shoulder joint, rather than at the elbow, because the shoulder joint experiences significantly larger torque loads compared to the elbow joint; 3) the calculated forces cannot exceed the maximal possible force values associated with the muscles (see Table 2). The above assumptions allow us to find one parameter family of solutions for the system (3) (a family of motor programs) for all six muscle forces. Parameter *d* defines the bi-articular muscles’ level of participation, such that *d* = 1 indicates that the torque at the shoulder is fully generated by the single-joint shoulder muscles, while *d* = 0 indicates that the torque at the shoulder is fully generated by the bi-articular shoulder muscles. In all the performed simulations, the torque distribution parameter *d* was randomly varied in the interval (0.5, 1) (unless otherwise stated), using a uniform probability distribution, to construct an ensemble of possible motor programs for every particular reaching movement.

### Motoneuron pool signals

Activities of six motoneuron populations (*MN*) corresponding to and controling the six muscles actuating the biomechanical arm is given by:

$$MN=\left(F/F_{max}-F_{p}\right)/\left(F_{l}\cdot F_{v}\right)$$ (15)

where *F* is the muscle force calculated in the previous step, and *F*_*max*_, *F*_*p*_, *F*_*l*_, and *F*_*v*_ are muscle specific parameters explained above (see Eq (4) and comments below the equation).

### Activities of cortical neurons

The six cortical neurons form the supra-spinal input drives ($\vec{MC}$) to the motoneurons. Calculation of their desired activity patterns is achieved by numerically inverting the dependence of motoneuron activity on cortical inputs (8) using the adapted secant method implemented as the following iterative procedure:

$$MC_{i+1}^{j}=MC_{i}^{j}-\left(Y_{i}^{j}-MN^{j}\right)\left(MC_{i}^{j}-MC_{i-1}^{j}\right)/(Y_{i}^{j}-Y_{i-1}^{j}),$$ (16)

where $j=\bar{1,6}$ is a component number, *i* is the iteration number, and $\vec{Y_{i}}=\vec{Y}({\vec{MC}}_{i},\vec{FB})$ (see Eq (8)). The map (16) converges to the solution of the inverse problem $\vec{Y}(\vec{MC},\vec{FB})=\vec{MN}$ concerned with finding the vector of cortical inputs $\vec{MC}$, such that the activity of spinal motoneurons is $\vec{MN}$ given the feedback $\vec{FB}$. All component of the feedback $\vec{FB}$ depend on the current state of the arm only, and are therefore fixed for every time step; and calculated using Eq (5).

### Simulation setup and regression analysis

Following many previous experimental studies, the movement direction was defined as the direction from the initial center position to a peripheral target position [2, 13, 14]. The 0^o^ direction is defined as moving the arm’s endpoint (wrist) to the right along a horizontal line, and moving the wrist in the opposite direction is the 180^o^ direction. Moving the wrist away from the body along a vertical line is the 90^o^ movement direction, and moving the wrist in the opposite direction is the 270^o^ movement direction. To understand the relationship between cortical activity and movement direction, our arm control model simulated several center-out reaching movements with an identical initial center position and equal reaching distances (radii) to 8 peripheral target positions in 8 different directions, at 45^o^ intervals from each other (Fig 2). The same type of center-out task has been used extensively in experimental studies [2, 13]. Average cortical activity, average muscle length and velocity, and average feedback signals were fitted to a cosine tuning curve using a regression function. The cosine tuning curve is given by:

$$f=b_{0}+b_{1}\;\sin\left(\theta \right){+b}_{2}\;cos(\theta ),$$

which is equivalent to:

$$f=b_{0}+c_{1}\;cos(\theta -\theta_{PD}),$$

where *b*_0_, *b*_1_, *b*_2_, and *c*_1_ are regression coefficients. *θ*_PD_ is the preferred direction in which the function has a maximal value. The coefficient of determination *R*^2^ was used to measure the degree of the regression fit. Note that the average of *f* across all directions (8 directions in this case) is equal to *b*_0_. The same regression analysis of cortical activity using a cosine tuning curve was previously used in several experimental studies [2, 13] to investigate the directional tuning of cortical neurons. The index of directional modulation, which describes the proportional increase or decrease over the mean activity level, is given by *I* = *c*_1_/*b*_0_.

For most of the center-out reaching tasks, except where noted, the following simulation parameters were used: integration time step of 0.001s; reaching time of 1s; reaching distance of 0.2m; and initial center position with Cartesian coordinates (0.0, 0.4). Note that we further investigated the center-out tasks with 16 different directions at 23^o^ intervals and found no significant differences in the obtained results. Therefore, we elected to use 8 directions for our study.

### Model validation

In order to evaluate the robustness of the model we independently varied basic parameters of the mechanical system (masses and lengths of arm segments) within ±15% range. In addition, we investigated the model’s behavior by varying maximal forces (*F*_*max*_) for each muscle in the same range (±15% around mean). For all simulations, there were no qualitative differences in the model’s performance. Specifically, the tuning curves of the cortical activities were similar to the corresponding tuning curves for the default values of the biomechanical variables. The statistical analysis based on the 50 simulations also did not show any significant differences in PDs for the chosen ranges of the basic biomechanical parameters (see Table 3).

**Table 3:** Variations in directional preferences of the cortical neurons and motoneurons due to variations in model parameters.

Neurons	PDs and R^2^ corresponding to different components											
	Sh-F		Sh-E		El-F		El-E		Bi-F		Bi-E	
	PD	R^2^	PD	R^2^	PD	R^2^	PD	R^2^	PD	R^2^	PD	R^2^
Cortical neurons	154.20±1.03	0.73±0.01	312.64±0.78	0.48±0.01	267.82±0.77	0.91±0.02	92.16±0.84	0.97±0.01	225.08±1.38	0.86±0.03	66.72±0.73	0.30±0.05
Moto-neurons	159.96±0.99	0.43±0.01	295.88±0.77	0.11±0.01	270.90±0.74	0.69±0.07	96.18±1.97	0.86±0.02	208.24±2.74	0.50±0.05	123.88±4.69	0.02±0.01

### Software

The model of the motor control system was implemented under MATLAB 8.4. The code used for the simulations presented here will be made available.

## Supporting information

S1 File Directional modulations and tuning curve fittings. The compressed zip file contains the data in ‘.mat’ format and the Matlab codes which can be accessed by MATLAB. The data and codes in this file produce reaching movement in 8 directions, directional modulation curves, tuning curves, preferred directions and coefficient of determinations.
(ZIP)
